# CD8 ^+^ T Cells Negatively Modulate Ischemia‐Induced Angiogenesis in Mice

**DOI:** 10.1096/fj.202503027R

**Published:** 2025-11-17

**Authors:** Xianji Piao, Jin Jingyuan, Longzhu Dai, Longguo Zhao, Yanglong Li, Megumi Narisawa, Shangzhi Shu, Yanna Lei, Xueling Yue, Jinshun Piao, Chengjie Zhu, Lina Hu, Qingsong Cui, Xian Wu Cheng

**Affiliations:** ^1^ Department of Intensive Care Unit (ICU) Yanbian University Hospital Yanji China; ^2^ Jilin Provincial Key Laboratory of Stress and Cardiovascular Disease Yanbian University Yanji China; ^3^ Department of Anesthesiology Yanbian University Hospital Yanji China; ^4^ Department of Oncology Yanbian University Hospital Yanji China; ^5^ Department of Cardiology Nagoya University Graduate School of Medicine Nagoya Japan; ^6^ Department of Cardiovascular Disease The First Hospital of Jilin University Changchun China; ^7^ Department of Public Health Guilin Medical College Guilin China; ^8^ Department of Cardiology and Hypertension Yanbian University Hospital Yanji China

**Keywords:** angiogenesis, CD8^+^ T cell, epigallocatechin‐3‐gallate, interferon‐γ

## Abstract

Angiogenesis‐related therapeutic approaches to peripheral arterial disease (PAD) deserve attention. CD8^+^ T cells play important roles in human pathobiology, and we investigated the involvement of cytotoxic CD8^+^ T cells in angiogenesis in response to ischemic stress. We also examined the mechanism of a component of green tea catechins, epigallocatechin‐3‐gallate (EGCG), which facilitates vascular regeneration in mice. Male 8‐week‐old wild‐type [(CD8a^+/+^) and interferon‐gamma (IFN‐γ^+/+^)] knockout (CD8a^−/−^ and IFN‐γ^−/−^) mice were subjected to unilateral hindlimb ischemic surgery and then loss‐of‐function studies by blood flow, molecular, and immunostaining analyses at several time points. Post‐ischemic surgery CD8a^+/+^ mice were treated with EGCG for the evaluation of its vasculoprotective effect. Ischemic stress increased CD8a^+^ T cells and IFN‐γ in blood and/or ischemic muscles. A serial laser Doppler blood‐flow analysis demonstrated a higher recovery of the ischemic/normal blood‐flow ratio in CD8a^−/−^ mice throughout the follow‐up period compared to CD8a^+/+^ mice. On postoperative day 14, CD8a^−/−^ ischemic muscles showed increased capillary density, vascular endothelial growth factor, p‐ERK1/2 and decreases in oxidative stress production and NLRP3 and caspase‐1 proteins, as well as the levels of matrix metalloproteinase‐2/9, cathepsin S/K, mac‐3^+^, and ssDNA^+^ cells in the ischemic muscles. All of these beneficial effects were reproduced in IFN‐γ^−/−^ mice. The vasculoprotection was diminished by the murine recombinant IFN‐γ supplementation. EGCG showed efficacy that was comparable to that of CD8a^−/−^ by modulating the growth signaling and apoptosis in mice. IFN‐γ depletion rescued impaired aortic ring angiogenic action. In human umbilical vein endothelial cells, EGCG ameliorated 5% T‐cell culture medium‐induced angiogenic actions, accompanied by reductions of NLRP3 and caspase‐1. Our findings indicate that CD8a^+^ T‐cell deficiency promotes angiogenesis, and EGCG can reverse the detrimental effects of CD8a^+^ T‐cell activation on angiogenesis. These results provide clinically relevant insights into the potential development of immune‐inflammatory therapy targeting vascular diseases.

## Graphical abstract

Graphical illustration of the mechanism of CD8^+^ T‐cells/IFN‐γ axis‐mediated angiogenesis in response to ischemic stress. CD8^+^ T‐cell functions as an important mediator of ischemia‐induced angiogenesis via the modulation of inflammation, oxidative stress, proteolysis, and endothelial proliferation that might be mediated by the IFN‐γ/NLRp3‐caspase‐1 and VEGF/Erk1/2 axes. And EGCG administration can improve ischemia‐induced angiogenesis by modulation of CD8^+^ T‐cells/IFN‐γ axis.

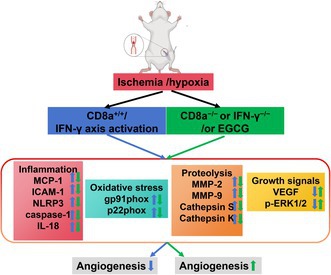

## 1 Introduction

Peripheral arterial disease (PAD) is characterized by a narrowing or blockage of arterial vessels that reduces the blood flow to the limbs, which is often caused by atherogenic lesions [1]. In severe cases of PAD, pronounced limb ischemia is observed, manifested as persistent foot pain at rest that is challenging to manage. The key pathophysiological changes include micro‐ and macrovascular dysfunctions that lead to lowered arterial perfusion and tissue hypoxia, which are often linked to multi‐segmental, diffuse, and progressive atherogenesis [2]. Hypoxic stress induces mature endothelial cell‐based angiogenesis and endothelial progenitor cell‐based vascularization, leading to the formation of ischemic tissue feeder collateral vessels and capillaries [3]. Therapeutic approaches with both multiple growth factors and stem cells have been extensively studied in the field of tissue regenetation [1, 4]. However, due to these approaches' limited clinical applications, an effective, safe, and cost‐efficient strategy to promote vascular regeneration is urgently needed.

Upon ischemic injury, new smaller microvessels and capillaries are regenerated through sprouting angiogenesis, during which immune cells may participate in the mechanisms of endothelial cell migration and proliferation as well as apoptosis [5]. CD8^+^ T cells, with known cytotoxic functions, have been demonstrated to modulate various aspects of tumor growth and tissue remodeling [6, 7]. One of the key effector molecules produced by these cells is interferon‐gamma (IFN‐γ), which plays a critical role in the induction of apoptosis in target cells. A disturbance of tumor‐induced angiogenesis by CD8^+^ T cells was closely associated with tumor rejection [8]. An adoptive transfer of tumor‐specific CD8^+^ T cells from IFN‐γ‐competent mice (IFN‐γ^+/+^) suppressed angiogenesis compared to the corresponding cells from IFN‐γ‐deficient mice (IFN‐γ^−/−^) in a mouse lung metastasis model [8]. Our group also showed that an adoptive transfer of CD8^+^ T cells of IFN‐γ^−/−^ mice ameliorated calcium chloride_2_‐induced abdominal aortic aneurysm formation in CD8a^+/+^ mice [9]. However, little is known about the CD8^+^ T/IFN‐γ axis in the modulation of ischemic angiogenesis, and its mechanisms remain largely unknown.

Emerging evidence suggests that epigallocatechin‐3‐gallate (EGCG), a natural antioxidant found predominantly in green tea, has the ability to modulate various cellular functions in the initiation and progression of proliferative diseases, including migration, invasion, proliferation, apoptosis, and immune responses [10, 11]. Based on its antioxidant and anti‐proteolytic functions, EGCG is commonly used for the prevention of tumors and cardiovascular diseases. Our group demonstrated that EGCG can inhibit vascular smooth muscle cell invasion and neointimal formation via the modulation of matrix metalloproteinase‐2 (MMP‐2) activity by the upregulation of its endogenase tissue inhibitor in a rat carotid artery injury model [12, 13]. EGCG may thus have value for improving the limb ischemic microenvironment [11]. We speculated that in this context, EGCG may be able to reverse the inhibitory effects of CD8^+^ T cell‐derived IFN‐γ on angiogenesis, offering a novel therapeutic approach for enhancing vascular regeneration.

We conducted the study to evaluate the impact of IFN‐γ‐secreting CD8^+^ T cells on angiogenesis and to investigate how EGCG exerts a vascular beneficial effect on angiogenesis in response to ischemic stress. Understanding these mechanisms could provide new insights into the interplay among immune regulation, metabolism, and vascular regeneration, with potential implications for developing targeted therapies in the management of patients with PAD.

## 2 Materials and Methods

### 2.1 Mice

All animal experimental procedures were performed according to the Guide for the Care and Use of Laboratory Animals published by the US National Institutes of Health and approved by the Institutional Animal Care and Use Committee of Yanbian University (protocol no: YD202309110014). Six‐week‐old male wild‐type mice (called CD8a^+/+^ or IFN‐γ^+/+^; C57BL/6 background) were purchased from Yanbian University Animal Center (Yanji, Jilin PR, China) and were used as the controls. CD8a^+^ T‐cell deletion (CD8a^−/−^) and IFN‐γ deletion (IFN‐γ^−/−^) mice (both C57BL/6 background) were created by the Shanghai Biomodel Organism Science & Technology Development Co. (Shanghai, China; Protocol no. 2021‐W5‐2174). Six‐week‐old male mice (18–23 g weight) were provided water ad libitum and food and housed under a 12 h light/12 h dark cycle for 2 weeks. The mice were monitored continuously during the experimental period, and all procedures were carried out by trained research personnel. Anesthesia and euthanasia procedures are detailed in each section.

### 2.2 Network Pharmacology Analysis

To identify potential therapeutic targets, we conducted a comprehensive search of the GeneCards database (https://www.genecards.org/), retrieving 5141 *Homo sapiens* targets associated with acute myocardial infarction (AMI). Additionally, epigallocatechin gallate (EGCG)‐related targets were obtained from multiple databases, including TCMSP (http://tcmspnw.com/), SwissTargetPrediction (http://swisstargetprediction.ch/), PharmMapper (http://www.lilab‐ecust.cn/pharmmapper/), and PubChem (https://pubchem.ncbi.nlm.nih.gov/), yielding 100 potential targets. Protein–protein interaction (PPI) networks for both AMI and EGCG were constructed using STRING databases (https://cn.string‐db.org/). Key hub nodes were identified by selecting intersection targets with degree values above the network average. Functional enrichment analysis of these key targets was performed using the DAVID platform (https://davidbioinformatics.nih.gov/summary.jsp), including Kyoto Encyclopedia of Genes and Genomes (KEGG) pathway analysis and Gene Ontology (GO) annotation. Bubble plots were generated in RStudio to visually represent the enrichment results, emphasizing significant biological processes and signaling pathways.

### 2.3 Hindlimb Ischemic Angiogenesis Induction and Treatment

Eight‐week‐old male mice were subjected to the sham and ischemic operations under anesthetization with 3% isoflurane inhalation in an induction chamber as described [14]. The mouse hindlimb ischemic model was induced by the ligation of the femoral artery at two points, distal and proximal, to the bifurcation of the deep femoral and superficial arteries, followed by removal of the intervening segment without injuring the nervus femoralis (Figure S1A) [15]. Following anesthetization with 3% isoflurane inhalation, mice that underwent a sham operation without femoral artery ligation were analyzed as the control group.

First, to examine the role of CD8^+^ T cells in ischemic angiogenesis (Exp. 1), CD8a^+/+^ and CD8a^−/−^ mice that were randomly assigned to the sham and ischemic operations were subjected to a blood flow evaluation and biological and immunohistological analyses at the indicated timepoints. Second, to examine the role of IFN‐γ in ischemic angiogenesis (Exp. 2), IFN‐γ^+/+^ and IFN‐γ^−/−^ mice that were randomly assigned to the sham and ischemic operations underwent the same evaluations as those mentioned above. Third, in the specific IFN‐γ administration experiments (Exp. 3), CD8a^−/−^ mice that underwent ischemic surgery were randomly assigned to two groups: the CD8a^−/−^‐saline group (Cont: intraperitoneally administered 100 μL of saline per mouse, 2×/week) and the CD8a^−/−^‐rIFN‐γ group [(rIFN‐γ: intraperitoneally given recombinant murine rIFN‐γ (cat.# P1015, APExBIO Technology, Houston, TX) at 50 000 U/100 μL per mouse, 2×/week; for 3 weeks (total six administrations))] and were subjected to the analyses. Figure S1 provides all experimental protocols.

Finally, to examine the vasculoprotective ability of EGCG (Exp. 4), wild‐type mice that underwent the ischemic surgery were randomly assigned to two groups: the Cont group administered 200 μL of saline per mouse by gavage and the EGCG group given 200 μL of EGCG solution (cat.#. 989‐51‐5, Sigma‐Aldrich Chemicals, St. Louis, MO) at 25 mg/kg/day. Both groups of mice were treated before ischemic surgery for 3 day (daily at 8:00–9:00 a.m.) and continuously until sacrifice before the evaluations and sampling.

At the indicated timepoints after the evaluation of the hindlimb blood flow, mice were euthanized by CO_2_ inhalation and were perfused through the left cardiac ventricle with phosphate‐buffered saline (PBS) [16]. For the biological analysis, the skeletal muscles was isolated and kept in RNAlater solution (gene assays) or liquid nitrogen (protein assays). For the immunostaining, the muscles were embedded in Tissue‐Tek optimal cutting temperature (OCT) compound (Sakura Finetechnical, Tokyo) and stored at −20°C.

### 2.4 Ischemic Hindlimb Perfusion Assay

Mice were anesthetized with 3% isoflurane inhalation in an induction chamber, and blood perfusion was evaluated by a laser Doppler blood flow (LDBF) imager (Lisca, Jarfalla, Sweden) on days 0, 4, 7, and 14 following ischemia [17]. To avoid data variations due to ambient temperature and light, the quantitative evaluation of the blood flow results is presented as the ratio of the left (ischemic) to the right (nonischemic) hindlimb.

### 2.5 Measurement of Capillary Density

The capillary density of the adductor muscle was evaluated with DyLight 594‐labeled *Lycopersicon esculentum* lectin (cat.# DL‐1177‐1, Vector Laboratories, Burlingame, CA) on postoperative day 14. The capillary endothelial cells were analyzed by the calculation of the lectin^+^ cells. Four randomly chosen microscopic fields from four to six different sections in each skeletal muscle were captured with an Evos microscope (Invitrogen, Carlsbad, CA) at 20× magnification.

### 2.6 Western Blot Analysis

The cultured cells or fresh skeletal muscles were lysed in RIPA lysis buffer containing protein phosphatase inhibitor cocktail and phenylmethylsulfonyl fluoride (Solarbio Life Sciences, Beijing, China) to extract the total protein [18]. The protein concentration of each sample was measured by a BCA protein assay kit (Solarbio Life Sciences), with a microplate reader's measurements taken at 562 nm. Twenty micrograms of total protein from each sample was separated by sodium dodecyl sulfate‐polyacrylamide gel electrophoresis (SDS‐PAGE).

After sequential transferring and blocking with Tris–HCl–buffered saline‐Tween containing 5% skim milk, the FluoroTrans‐W membranes (Pall, Port Washington, NY) were treated with the first antibodies as follows: nod‐like receptor family pyrin domain‐containing 3 (NLRP3, #15101, 1:1000), caspase‐1 (#24232, 1:1000), phospho‐extracellular signal‐regulated kinase 1/2 (p‐Erk1/2, #4377, 1:1000), Erk1/2 (#4695, 1:1000), glyceraldehyde 3‐phosphate dehydrogenase (GAPDH, #5174, 1:1000; all from Cell Signaling Technology, Danvers, MA), and vascular endothelial growth factor‐A (VEGF‐A, ab46154, 1:1000; Abcam, Cambridge, MA). The membranes were then treated with the secondary antibodies (anti‐mouse #14709, anti‐rabbit #14708, 1:5000–1:10 000; Cell Signaling Technology) for 2 h at room temperature. The targeted molecule bands were visualized using chemiluminescent substrates (Merck Millipore, Darmstadt, Germany). The targeted molecule band intensities expressed from western blots were normalized by loading the internal control GAPDH band.

### 2.7 Evaluation of the Plasma VEGF and IL‐18 Levels

The mouse plasma levels of VEGF (cat.# MMV00, R&D Systems, Minneapolis, MN), interleukin (IL)‐18 (cat.# ml‐1066842), and IFN‐γ (cat.# ml‐002277; both from MLBIO, Shanghai, China) were examined using enzyme‐linked immunosorbent assay (ELISA) kits [19].

### 2.8 Oxidative Stress Production Assay

We used a lucigenin‐based enhanced chemiluminescence assay to evaluate the reactive oxygen species (ROS)–generating enzyme nicotinamide adenine dinucleotide phosphate (NADPH) oxidase‐dependent superoxide production of the fresh skeletal muscle tissues, as described [20]. In brief, 1 mL of the muscle homogenate protein was applied to an assay tube. The chemiluminescence signal was measured every 60 s for 12 min with a luminometer (Tuner Designs, Sunnyvale, CA), and the respective background counts were subtracted from the experimental values as described previously [21].

### 2.9 Evaluation of Macrophage Infiltration

The infiltration of macrophages was evaluated by an immunofluorescence analysis [9]. The skeletal muscle was fixed in 4% paraformaldehyde and embedded in the Tissue‐Tek OCT Compound, and 5 μm sections were cut and mounted on MSA‐coated slides. The corresponding sections on separate slides were blocked with 5% goat serum. The sections were then treated with fluorescein isothiocyanate‐labeled CD68 monoclonal antibody (1:200, bs‐33056 m, Bioss, Woburn, MA). Four to six images from each section at a 40× objective were used to calculate the numbers of CD68^+^ cells and average the numbers for each mouse.

### 2.10 Gelatin Zymography

Total proteins extracted from the muscles obtained on day 4 postsurgery were mixed with SDS sample buffer with a non‐reducing agent and loaded onto the 10% SDS‐polyacrylamide gel containing 1 mg/mL gelatin. The gels were incubated in MMP reaction buffer (1% Triton X‐100, 5 mol/L CaCl_2_, 1 μmol/L ZnCl_2_, 50 mmol/L Tris–HCl, pH 7.5) at 37°C overnight. Each gel was then stained with Coomassie Brilliant Blue for 60 min, followed by treatment in a destaining solution until the bands became clearly visible [9].

### 2.11 Targeted Gene Assay

Total RNA was extracted from the lysates of the muscle tissues following the Trizol protocol (Ambion, Carlsbad, CA) and then reverse‐transcribed to cDNA using a reverse transcriptase kit (ZOMANBIO, Beijing) [22]. A quantitative polymerase chain reaction (qPCR) was applied to analyze the resulting cDNA with an ABI 7300 real‐time PCR system (Applied Biosystems, Foster City, CA) with specific primers for cathepsin K (*CatK*), *CatS*, *MMP‐9*, *MMP‐2*, intercellular adhesion molecule‐1 (*ICAM‐1*), monocyte chemotaxis protein‐1 (*MCP‐1*), gp91^phox^, *and* p22^phox^. These investigated gene levels were normalized to *GAPDH* mRNA levels. All experiments were performed in triplicate with specific primers. The primer sequences of all genes are described in Table S1.

### 2.12 Cell Apoptosis Assay

On day 4 postsurgery, the corresponding sections were preincubated with 5% serum and then incubated with the primary antibody against single‐stranded DNA (ss‐DNA; 1:200; Dako, Glostrup, Denmark). Immunostaining was visualized using an ABC kit (Vector Laboratories) [23]. Levamisole (1:100; Vector Laboratories) was used as an endogenous alkaline phosphatase inhibitor. Four randomly chosen microscopic fields from four to six different sections in each skeletal muscle were captured with the Evos microscope at 20× magnification for the ssDNA^+^ cell calculation and averaged for each animal.

### 2.13 Aortic Ring Culture Assay

An ex vivo angiogenesis assay was performed as described [17]. The rings obtained from the thoracic aortas of wild type (CD8a^+/+^ and IFN‐γ^+/+^) mice were seeded onto type I collagen gels (BD Biosciences, San Jose, CA) and cultured in endothelial cell basal medium‐2 (EBM‐2, Chambrex, East Rutherford, NJ) in the presence of 50 ng/mL VEGF (abs01017, Absin, Shanghai, China) for 7 days. Sprouted microtubes were characterized by an immunofluorescence fluorescein isothiocyanate‐labeled mouse anti‐CD31 antibody (BD Pharmingen, San Diego, CA). Sprout lengths were measured using confocal microscopy with the Evos microscope at 200× magnification. The densities of the sprouted endothelial cells are presented as the percentage of pixels per image occupied by each aortic ring within a defined area.

### 2.14 Cell Culture

We performed naïve CD8^+^ T‐cell activation as described [24]. In brief, naïve CD8^+^ T cells (FuHeng Biology, Shanghai, China) were activated in vitro with a T‐Cell Activation/Expansion Kit (cat.# 130‐091‐441, Miltenyi Biotec, Bergisch Gladbach, Germany) for 3 days. Following activation, CD8^+^ T lymphocytes were expanded for 5 days in the presence of 20 units/mL recombinant murine IL‐2 (cat.# 130‐097‐745, Miltenyi Biotec) for 3 days. Next, human umbilical vein endothelial cells (HUVECs; FuHeng Biology) were cultured with 5% naïve CD8^+^ T cell‐conditioned medium (NTCM) plus human recombinant VEGF (rhVEGF, 20 ng/mL; cat.# abs05164, absin) or 5% activated T cell‐conditioned medium (ATCM) plus rhVEGF (20 ng/mL), respectively, under hypoxic conditions for 24 h and then subjected to biological analyses.

### 2.15 Tube Formation

For the evaluation of tube formation, 2.5 × 10^5^ HUVECs were seeded onto Matrigel (BD Biosciences) in a 24‐well plate and cultured for 24 h with 5% NTCM containing 20 ng/mL of rhVEGF, 5% ATCM containing 20 ng/mL of rhVEGF, 5% ATCM containing 20 ng/mL of rhVEGF, and 20 μmol/L of EGCG under hypoxic conditions to induce microtube formation. Microtube lengths were evaluated using Image J with the Angiogenesis Analyzer plugin in six fields (100×) of each well.

### 2.16 Cell Migration Assay

The cell migration assay used the Transwells of 24‐well plates (Costar, Cambridge, MA) composed of a polycarbonate membrane with 5‐μm pores as described [25]. The membrane was coated with 50 μL of Matrigel solution (50 μg/mL, Becton Dickinson) for 12 h at 4°C, then rinsed well with PBS three times. HUVECs were seeded in the inner chamber of the Transwell at 5 × 10^4^ HUVECs cells in 100 μL of 5% NTCM or 5% ATCM. For the assessment of the effects of EGCG, the HUVEC suspension containing EGCG (0 or 20 μmol/L) was plated in the inner chamber. The inner chamber was placed into the outer chamber, which contained 600 μL of 5% NTCM containing 20 ng of rhVEGF or 5% ATCM containing 20 ng/mL of rhVEGF and EGCG at the indicated concentrations (0 and 20 μmol/L), and it was then incubated for 5 h at 37°C in a hypoxic CO_2_ incubator. The HUVECs that migrated to the outer side of the membranes were stained and counted in 5–7 randomly chosen fields of the duplicate chambers at ×200 magnification for each sample.

### 2.17 Flow Cytometry Analyses of CD4 ^+^ and CD8 ^+^ Cells and Apoptosis

On postoperative day 4, after the lysing and fixing of peripheral blood (200 μL) from the experimental groups, the cell results were incubated with an APC‐labeled rat anti‐mouse CD4^+^ (FACS 553 051, BD Pharmingen) and PE‐labeled rat anti‐mouse CD8^+^ for 40 min and then subjected to flow cytometry as described [26]. In addition, following spleen homogenizing, the results were subjected to flow cytometry used APC‐labeled rat anti‐mouse CD44 (CD44‐559250, BD Pharmingen) and PE‐labeled rat anti‐mouse CD64L (CD62L‐553151, BD Pharmingen) for the evaluations of spleen CD44^high^CD64L^low^ CD8a^+^ T‐cell subset (called effector memory T cells).

For the assessment of the effects of the activated CD8^+^ T cell‐cultured medium on endothelial cell apoptosis, we used a fluorescein isothiocyanate (FITC) Annexin V Apoptosis Detection Kit (cat.# 640914, Biolegend, San Diego, CA) with a propidium iodide (PI) double stain assay for cell apoptosis per the manufacturer's protocol.

In brief, following culture with 5% NTCM, 5% ATCM, and 5% ATCM containing 20 μM of EGCG, respectively, the HUVECs were collected and resuspended in 100 μL of binding buffer including 1 μL of PI and 1 μL of annexin V‐FITC and then incubated for 15 min at room temperature in the dark. The apoptotic cell evaluation was performed immediately with a flow cytometer.

### 2.18 Statistical Analysis

All data are expressed as the mean ± standard error (SE). A one‐way analysis of variance (ANOVA) (for comparisons of three or more groups), followed by Tukey's post hoc test or Student's *t* test (for comparisons of two groups) was conducted. Blood‐flow data were subjected to two‐way repeated‐measures ANOVA and Bonferroni's post hoc tests. The length and number of endothelial sprouts and collateral capillary density were examined by two observers in a blind manner, and the values they obtained were averaged. GraphPad Prism ver. 8.0.2 was used. A value of *p* < 0.05 was considered significant.

## 3 Results

### 3.1 Network Pharmacology Analysis to Identify Targeted Molecules in the Ischemic Myocardium of the Mice With Acute Myocardial Infarction (AMI)

**FIGURE 1:**
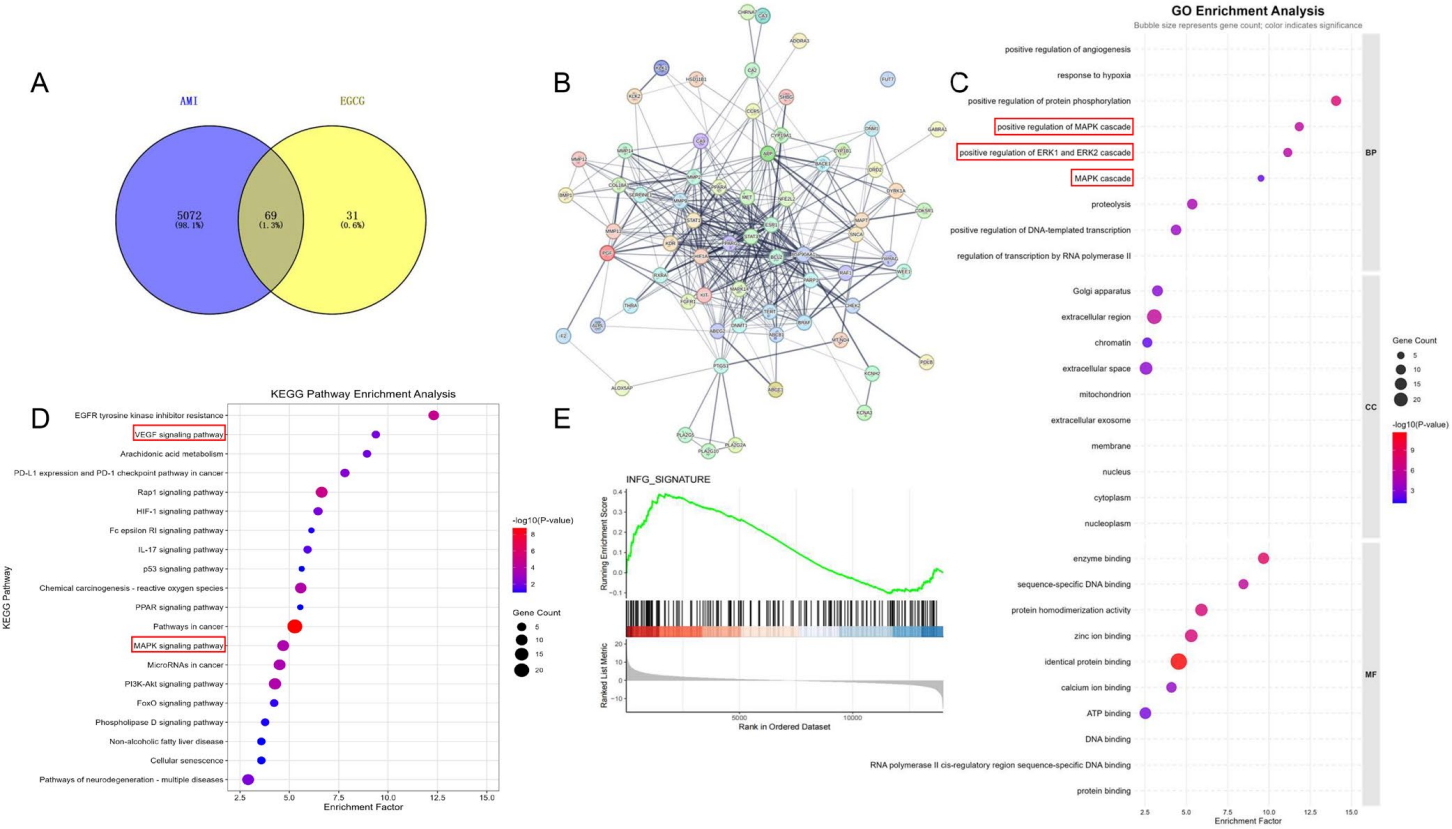
Network pharmacology combined with RNA‐seq analysis indicates that AMI activates the Keap1‐Nrf2‐HO‐1. (A) 5141 AMI associated targets and 100 EGCG‐related targets were collected from free‐access databases. Merging these networks revealed 69 common genes, potential targets for EGCG in AMI prevention. (B) Protein–protein interaction network for 69 target genes through the STRING database. (C, D) Functional enrichment analysis using KEGG and GO databases identified key biological pathways and processes related to the 69 potential therapeutic targets. (E) GSEA (Gene Set Enrichment Analysis) on the differentially expressed genes from GSE228871 by RStudio and found that the IFN‐γ signaling pathway was significantly enriched.

To investigate the potential molecular targets of EGCG treatment in AMI mice, a network pharmacology analysis was conducted. A total of 100 genes associated with EGCG treatment were identified using the SwissTargetPrediction database, while 5141 genes related to AMI were retrieved from the GeneCards database. Subsequently, a Venn diagram was constructed using Venny 2.1.0, revealing 69 overlapping targets (Figure 1A). To visually evaluate the EGCG intervention on AMI, the 69 common targets were imported into the STRING database to construct a protein–protein interaction (PPI) network (Figure 1B).

To systematically explore the potential mechanisms of EGCG treatment in AMI, we performed KEGG pathway analysis and GO enrichment analysis to identify the signaling pathways associated with EGCG's potential therapeutic targets in AMI. We performed KEGG and GO enrichment analysis on these 69 common targets using the DAVID database. Among the identified pathways, we presented 20 KEGG pathways including the VEGF signaling pathway, and 30 GO terms including the positive regulation of the positive regulation of ERK1 and ERK2 cascade (Figure 1C,D). Subsequently, we conducted differential analysis of the injured muscles in AMI mice from GSE228871 by RStudio. We also performed GSEA (Gene Set Enrichment Analysis) on the differentially expressed genes and found that the IFN‐γ signaling pathway was significantly enriched (Figure 1E). These findings suggest that EGCG might produce a benefit on AMI through the VEGF‐Erk1/2 signaling pathways. IFN‐γ is also involved in the pathogenesis of AMI. As known, vascular regeneration is a critical process in cardiac repair following AMI, and these data provide a theoretical foundation for further mechanistic exploration of vascular regeneration.

### 3.2 CD8 Deletion Improved the Blood Flow Recovery and Capillary Formation in Response to Ischemic Stress

**FIGURE 2:**
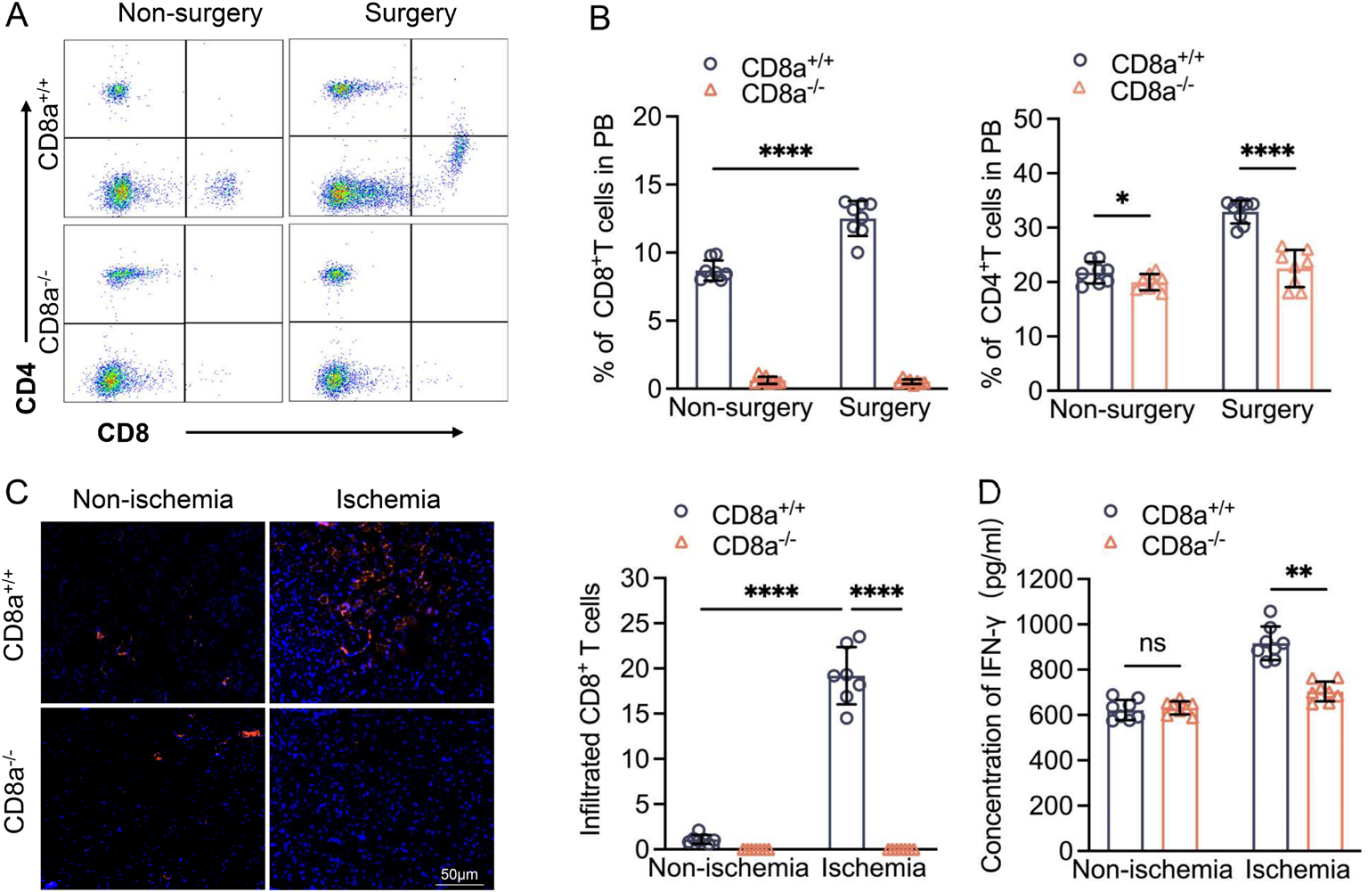
Ischemic stress increased circulating CD8^+^ T cells and their infiltration into the ischemic muscles in CD8a^+/+^ mice. (A) Gating for the enclosed two populations isolated from peripheral blood (PB) cells of four groups and for identifying CD4^+^ T cells and CD8^+^ T cells in CD8a^+/+^ mice (*upper panels*) and CD8a^−/−^ mice (*bottom panels*). (B) PB CD8^+^ T‐cell and CD4^+^ T‐cell numbers in both genotypes of mice at 1 week of ischemic induction. (C) Representative immunofluorescence images and quantitative data showing the numbers of infiltrated CD8^+^ T cells in ischemic and nonischemic muscles. (D) ELISA results showing the levels of serum IFN‐γ in the four groups. Data are mean ± SEM (*n* = 7–8/group). Significance was assessed by one‐way ANOVA, followed by Tukey's post hoc tests (B, D, E). *p < 0.05, **p < 0.01, ****p < 0.0001. Scale bar, 50 μm.

**FIGURE 3:**
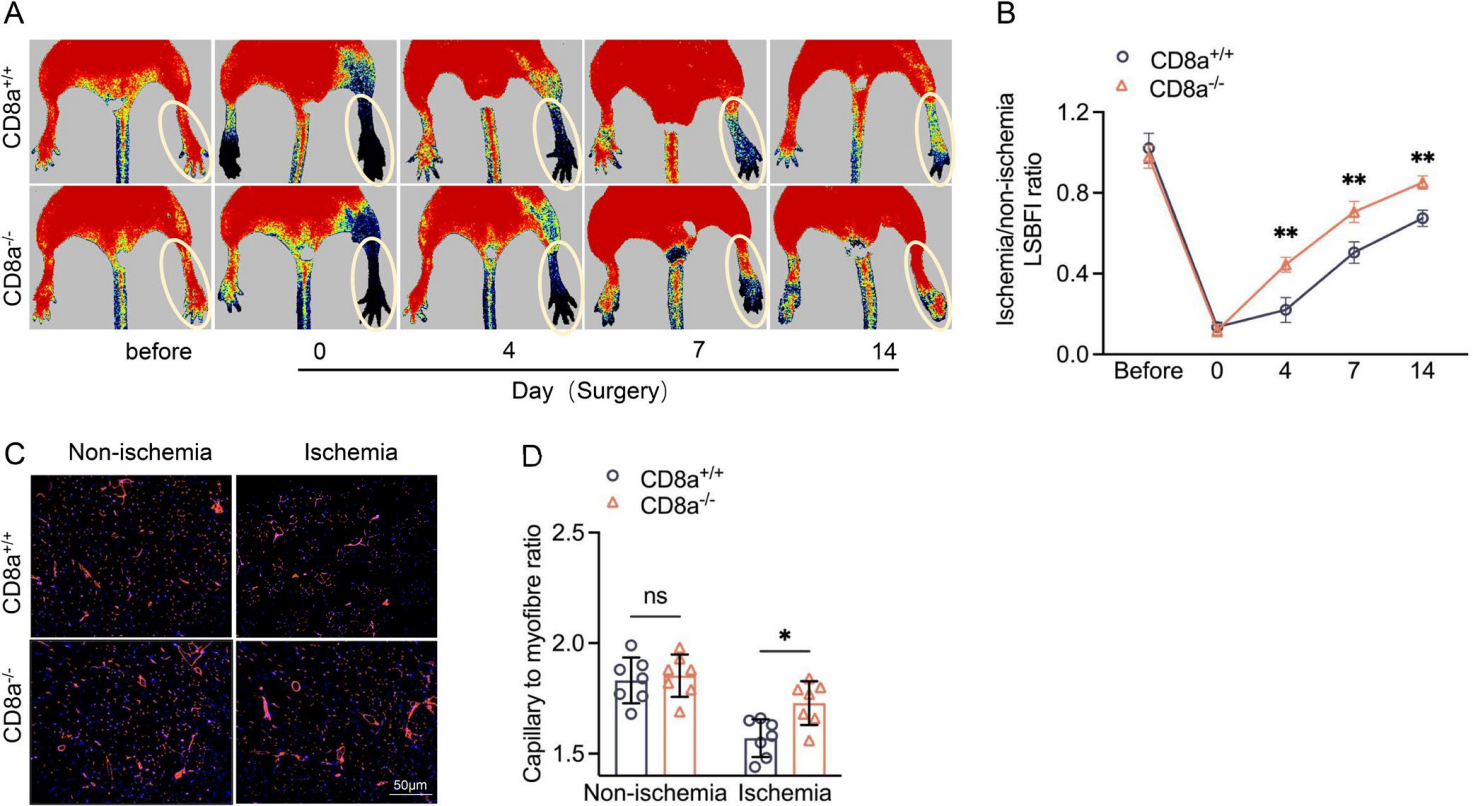
CD8a^−/−^ mice exhibited enhanced ischemic muscle blood flow recovery and capillary formation. (A, B) Representative laser Doppler blood flow (LDBF) images and quantitative data showing the ischemia‐to‐nonischemia blood flow ratio in the ischemic hindlimbs of CD8a^+/+^ and CD8a^−/−^ mice. (C, D) Immunofluorescence images and quantitative data show the capillary densities in ischemic and nonischemic thigh adductor muscles (*blue* = DAPI, *red* = tomato lectin‐Dylight‐594). Data are mean ± SEM (*n* = 7–8/group). Statistical significance was assessed by two‐way repeated measures ANOVA (B) or one‐way ANOVA (D). NS: Not significant. *p < 0.05, **p < 0.01. Scale bar, 50 μm.

To explore the role of CD8^+^ T cells in ischemia‐induced angiogenesis, we subjected CD8a^+/+^ and CD8a^−/−^ mice to a unilateral hindlimb ischemic angiogenesis model. As shown in Figure 2A–C, the CD8^+^ T cells were very sensitive to the induction by ischemic stress in the peripheral blood and ischemic muscles. The serial laser LDBF data showed that CD8^+^ T‐cell deletion improved the recovery of ischemic hindlimb blood perfusion throughout the follow‐up period, and the ratio of ischemic (left) to normal (right) LDBF was persistently higher in the CD8a^−/−^ mice compared to the CD8a^+/+^ mice (Figure 3A,B). On postsurgery day 14, the CD8^+^ T cell‐lacking mice had well‐developed capillaries in the ischemic muscles (Figure 3C,D), indicating that CT8^+^ T cells are crucial for neovascularization in response to ischemic stress. We also observed elevated CD4^+^ T cells in the blood in both genetic mice (Figure 2A,B).

### 3.3 CD8 ^+^ T‐Cell Activation Disturbed the Angiogenesis Associated With Lowered VEGF/Erk1/2 Signaling and Immune Inflammation–Oxidative Stress‐Mediated Apoptosis Axis Overaction in Mice Under Ischemic Conditions

**FIGURE 4:**
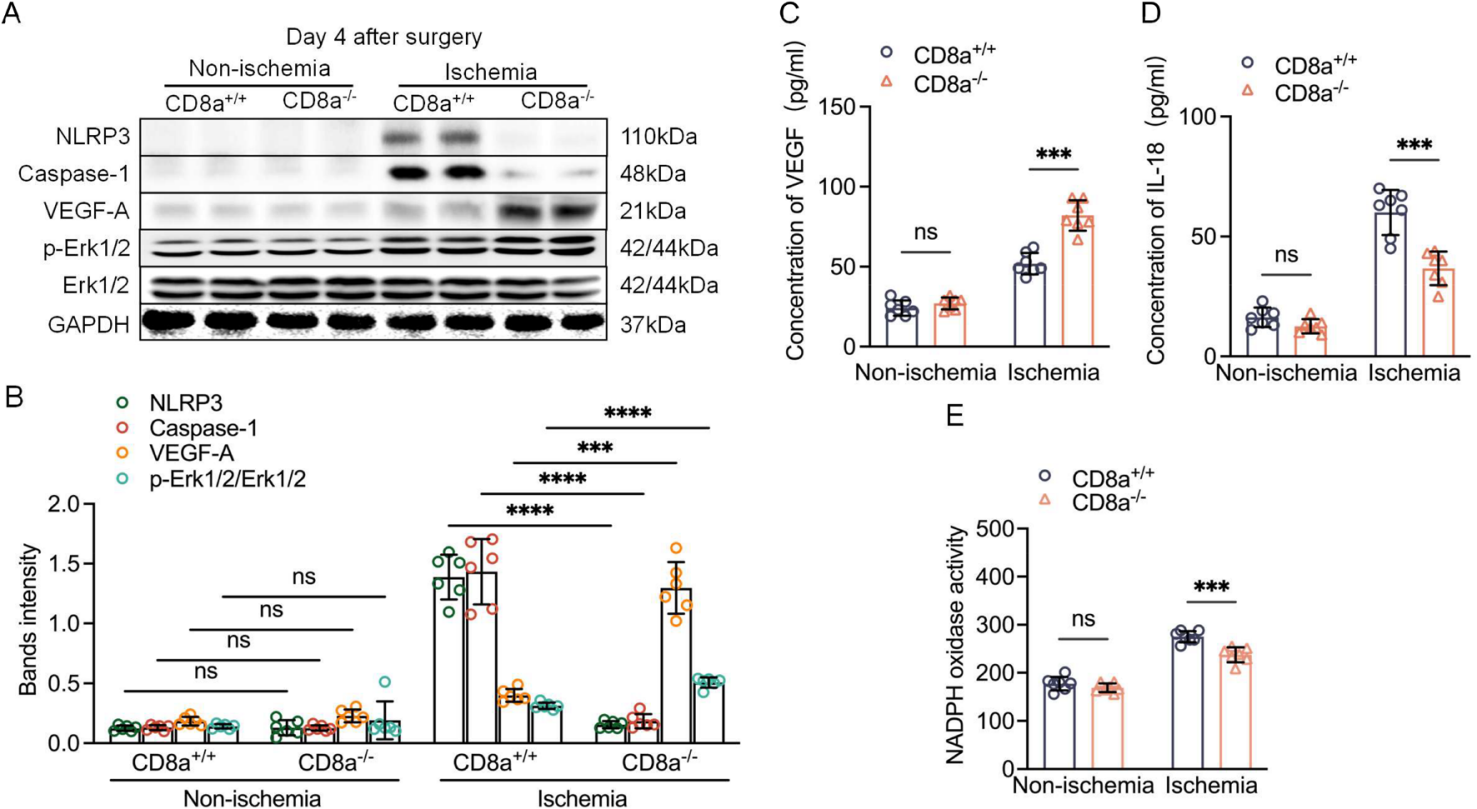
CD8a^−/−^ improved the growth signal‐ and pyroptosis‐related protein changes. (A, B) Representative western blotting images and quantitative data for the levels of NLRP3, caspase‐1, VEGF, and p‐Erk1/2 proteins in four groups (*n* = 6/group). (C, D) ELISA results showing serum VEGF and IL‐18 levels in the four groups (*n* = 7/group). (E) Nicotinamide adenine dinucleotide phosphate (NADPH) oxidase activity in homogenates of the ischemic skeletal muscle tissue (*n* = 7/group). Data are mean ± SEM. Significance was assessed by one‐way ANOVA followed by Tukey's post hoc tests (B–E). ***p < 0.001, ****p < 0.0001.

VEGF acts as a key modulator in vascular regeneration of various pathophysiological conditions [27]. On post‐ischemia day 14, representative images and the combined quantitative immunoblotting data demonstrated that the ischemic muscles had elevated levels of VEGF and its downstream Erk1/2 phosphorylated proteins in the CD8a^+/+^ mice, and this signaling activation was augmented in the CD8a^−/−^ mice (Figure 4A,B). The ELISA yielded the same conclusion regarding plasma VEGF levels (Figure 4C), suggesting that the vasculo‐protective actions of CD8^+^ T‐cell deletion are mediated, at least in part, through the VEGF‐dependent Erk1/2 signaling activation.

As a key component of the inflammasome, NLRP3 (a critical intracellular danger sensor [28]) was significantly upregulated by acute ischemic stress but negated by CD8^+^ T‐cell deficiency (Figure 4A,B). Likewise, in the muscles of the CD8a^+/+^ mice, ischemic injury caused an elevation of caspase‐1 protein, and this upregulation was less pronounced in the mice devoid of CD8^+^ T cells. The CD8a^−/−^ mice showed markedly lowered levels of blood IL‐18 and IFN‐γ protein, muscle MCP‐1 and ICAM‐1 gene expressions, and infiltrated macrophages compared to the CD8a^+/+^ mice (Figures 2D, 4D, S2A,B, S3A), which suggests that CD8^+^ T cell‐mediated NLRP3 inflammasome activation by ischemia could present a common mechanism in the deterioration of vascular regeneration in mice.

NADPH oxidase is present primarily in phagocytes such as neutrophils and macrophages [29]. The present ischemic stress resulted in a significant increase in the muscle NADPH activity and ssDNA^+^ apoptotic cells of the CD8a^+/+^ mice; these changes were reversed by CD8^+^ T‐cell deletion (Figures 4E and S4A,B). The qPCR analyses yielded the same conclusions regarding the NADPH oxidase subunit (gp91^phox^ and p22^phox^) gene expressions (Figure S3B). Because NADPH oxidase activity can act as a signaling molecule to promote inflammatory responses and apoptosis [30, 31, 32], we propose that CD8^+^ T cells might trigger the activation of the NADPH oxidase‐mediated NLRP3 inflammasome, playing a role in the immune–inflammation–apoptosis axis activation during angiogenesis following ischemia.

On the other hand, oxidative stress and inflammatory cytokines can regulate MMP and cathepsin expressions to contribute to neovascularization in human and animal pathologies [17, 33, 34]. Our present results revealed that the CD8a^−/−^ ischemic muscles had greatly reduced gelatinolytic activities of both MMP‐2 and MMP‐9 (Figure S2C,D). Consistent with these gelatin zymography data, the qPCR data confirmed that CD8a^−/−^ significantly lowered proteolysis‐related enzyme (MMP‐2, MMP‐9, cathepsin S, and cathepsin K) gene expressions in the ischemic muscles compared to the CD8a^+/+^ ischemic muscles (Figure S3C,D). Collectively, these findings suggest that CD8a^−/−^‐mediated vasculoprotection might be due to the activation of VEGF/Erk1/2 growth signaling and reductions of apoptosis and proteolysis that were associated with a lowered immune‐inflammation‐oxidative stress response.

### 3.4 IFN‐γ Deficiency Facilitated the Restoration of Vascular Regeneration in Response to Ischemia

**FIGURE 5:**
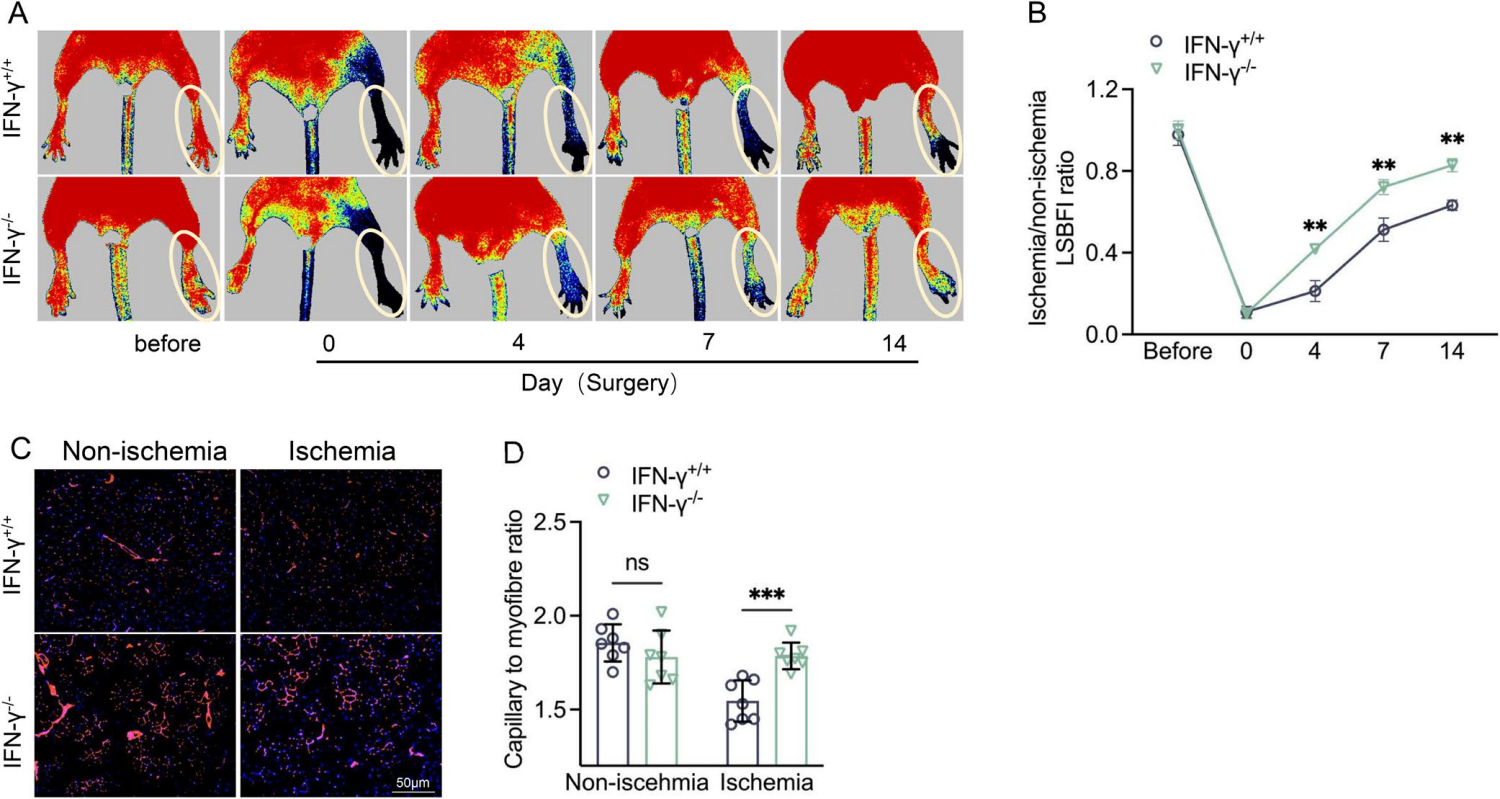
IFN‐γ deficiency facilitated ischemic muscle blood flow recovery and capillary formation. (A, B) Representative LDBF images and quantitative data showing the ischemia‐to‐nonischemia blood flow ratio in the ischemic hindlimbs of IFN‐γ^+/+^ and IFN‐γ^−/−^ mice. (C, D) Immunofluorescence images and quantitative data showing the capillary density in ischemic and nonischemic thigh adductor muscles. Data are mean ± SEM (*n* = 7–8/group). Significance was assessed by two‐way repeated measures ANOVA (B) or one‐way ANOVA (D, E). **p < 0.01, ***p < 0.001. Scale bar, 50 μm.

To further investigate possible mechanisms of CD8^+^ T cells in vascular regeneration, we studied a cytokine, IFN‐γ, that is secreted mainly by activated CD8^+^ T cells and can further activate an adaptive immune–inflammation response during angiogenesis under pathological conditions [35]. We examined the consequences of IFN‐γ deficiency in post‐ischemic angiogenesis. Similar to the CD8^+^ T‐cell knockout mice, the genetic ablation of IFN‐γ expression markedly facilitated ischemic muscle blood perfusion recovery during the follow‐up period and capillary formation at day 14 after injury (Figure 5), which leads us to propose that IFN‐γ might be derived from activated CD8^+^ T cells to act as an important mediator of ischemia‐induced angiogenesis.

**FIGURE 6:**
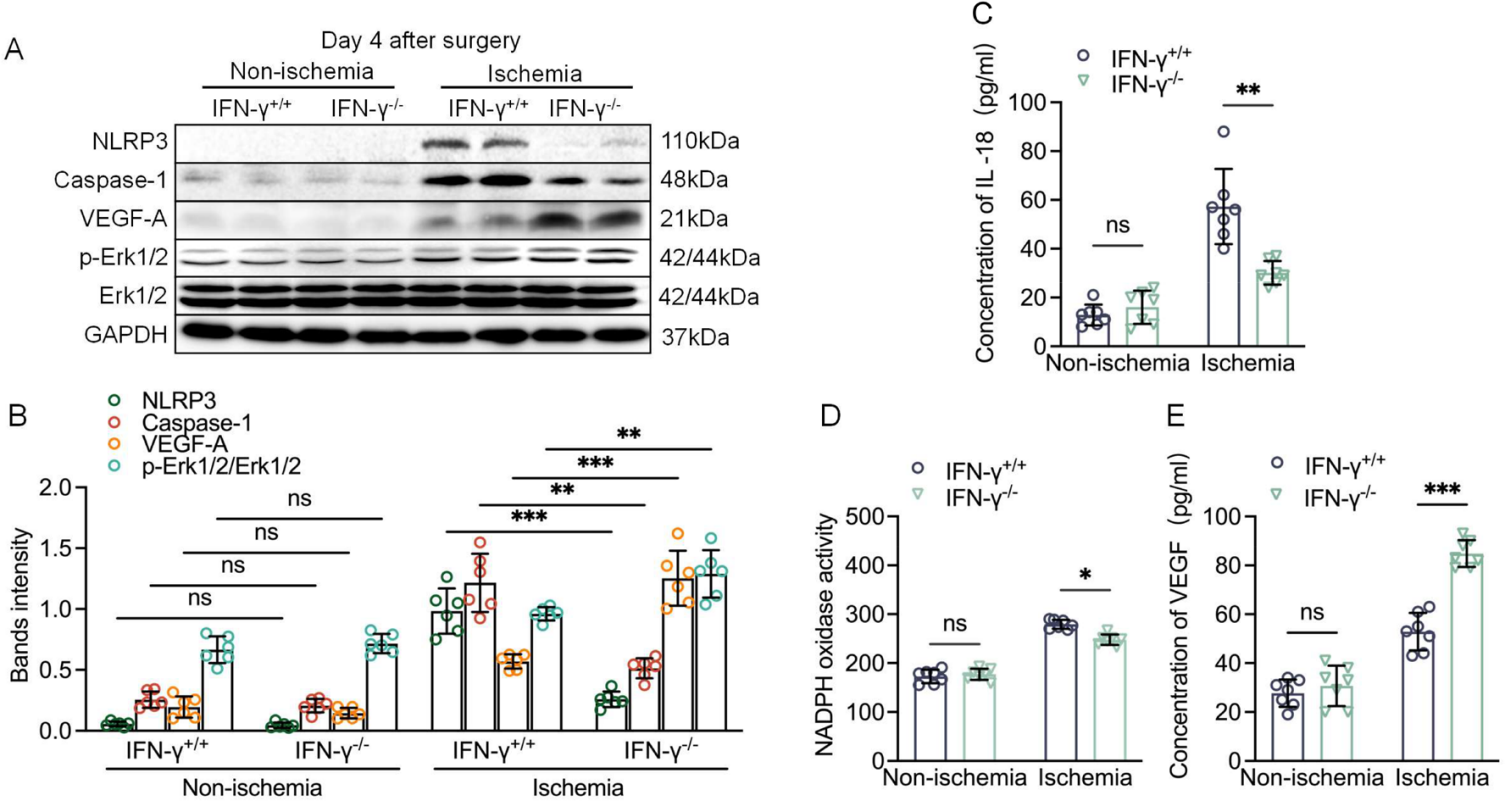
IFN‐γ deficiency improved growth signal‐ and pyroptosis‐related protein changes. (A, B) Representative western blotting images and quantitative data for the levels of NLRP3, caspase‐1, VEGF, and p‐Erk1/2 proteins in four experimental groups (*n* = 6/group). (C) ELISA results showing the serum levels of IL‐18 in four groups (*n* = 7/group). (D) NADPH oxidase activity in the ischemic skeletal muscles of the four groups (*n* = 7/group). (E): ELISA results showing the serum levels of VEGF in four groups (*n* = 7/group). Data are mean ± SEM. Significance was assessed by one‐way ANOVA followed by Tukey's post hoc tests (B–D). *p < 0.05, **p < 0.01, ***p < 0.001.

These results aligned with those of the CD8a^−/−^ mice, in which the NLRP3 and caspase‐1 levels were significantly decreased in the ischemic muscles of IFN‐γ^−/−^ mice compared to the IFN‐γ^+/+^ mice (Figure 6A,B). As anticipated, we also observed a significant reduction in plasma IL‐18 levels (Figure 6C). These results revealed that the MMP‐2 and MMP‐9 gelatinolytic activities and NADPH oxidase activity were higher in the ischemic muscles of IFN‐γ^+/+^ mice, and these effects were rectified by IFN‐γ deletion (Figures 6D and S5A,B). We also noted fewer ssDNA^+^ apoptotic cells and infiltrated macrophages in the ischemic muscles of the IFN‐γ^−/−^ mice compared to the IFN‐γ^+/+^ mice (Figure S5C–E). Consistently, IFN‐γ^−/−^ markedly lowered the levels of inflammation‐ (MCP‐1 and ICMA‐1), oxidative stress‐ (gp91^phox^ and 22^phox^), and proteolysis (MMP‐2, MMP‐9, cathepsin S, and cathepsin K)‐related genes in the ischemic muscles (Figure S6).

In contrast, the IFN‐γ^−/−^ mice exhibited remarkably upregulated growth signaling‐related proteins (VEGF and p‐Erk1/2) in the ischemic muscles and/or blood (Figure 6A,B,E). We also observed an increased IFN‐γ level in the IFN‐γ^+/+^ mice compared to the control mice, but not in the IFN‐γ^−/−^ mice (Figure S7A). Collectively, the upregulation of IFN‐γ by CD8^+^ T‐cell activation could represent a common mechanism in the deterioration of vascular regeneration capacity in response to ischemic stress.

### 3.5 Administration of Mouse Recombinant IFN‐γ Diminished CD8a ^−/−^‐Mediated Vasculoprotection

**FIGURE 7:**
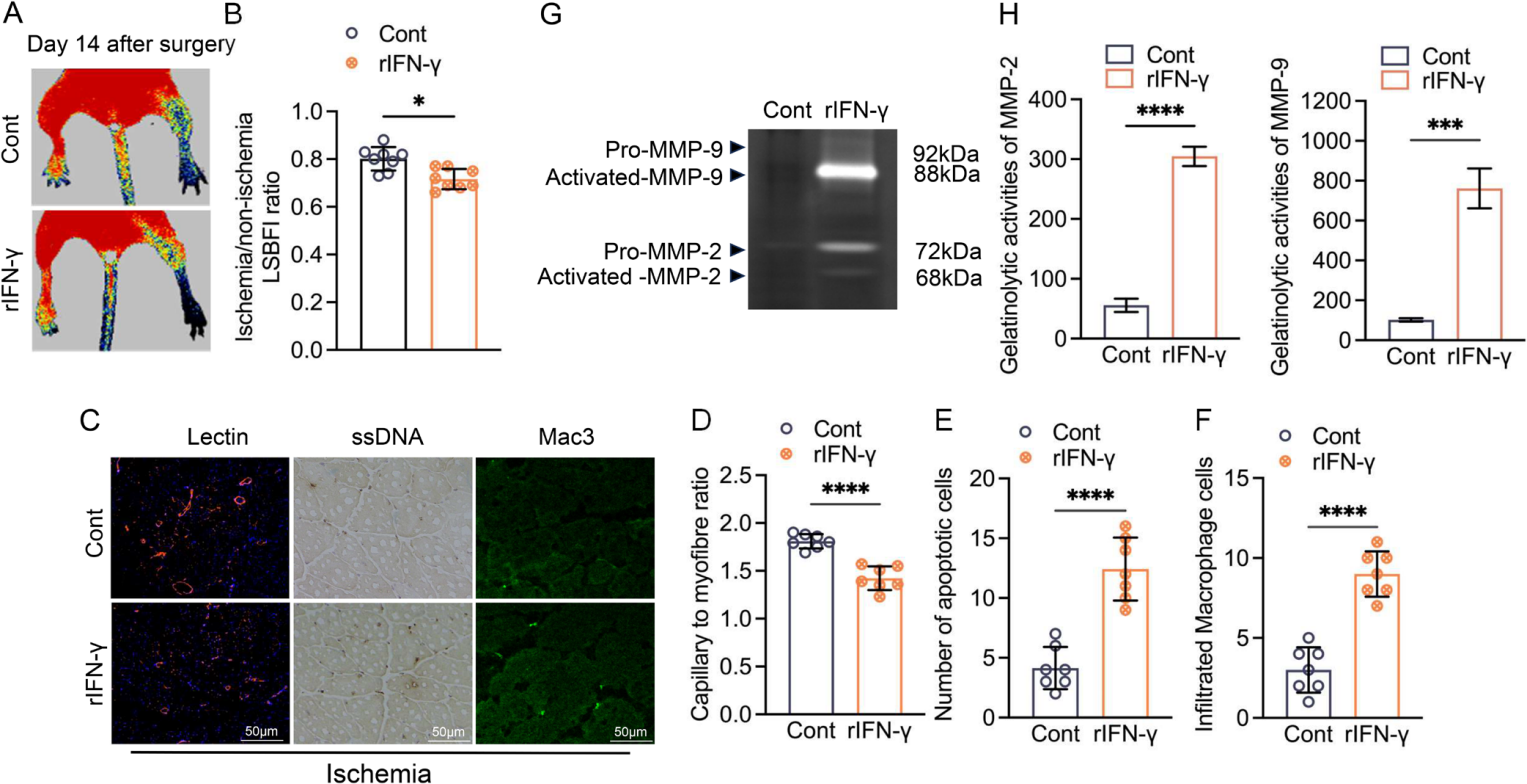
Murine recombinant IFN‐γ administration reduced the vascular regeneration in CD8a^−/−^ mice. (A, B) Representative LDBF images and quantitative data showing the ischemia‐to‐nonischemia blood flow ratio in the ischemic hindlimbs of CD8a^−/−^ + Saline and CD8a^−/−^ + rIFN‐γ mice (*n* = 8/group). (C–F) Representative staining images and quantitative data showing the numbers of lectin^+^, ssDNA^+^, and Mac3^+^ cells in the ischemic muscles of two experimental groups (*n* = 7/group). (G, H) Representative gelatin zymography images and quantitative data for the MMP‐2 and MMP‐9 gelatinolytic activities in two groups (*n* = 4/group). Data are mean ± SEM. Significance was assessed by unpaired Student's t‐test (B, D–H). *p < 0.05, ***p < 0.001, ****p < 0.0001. Scale bar, 50 μm.

To further explore the relationship between CD8^+^ T‐cell activation‐mediated impaired vascular regeneration capacity and IFN‐γ induction under our experimental conditions, CD8a^−/−^ mice that had undergone ischemic surgery were intraperitoneally administered recombinant murine IFN‐γ (50 000 U/100 μL per mouse, 2×/week) for 3 weeks and then subjected to the analyses at indicated timepoints. On day 14 after the induction of ischemia, CD8a^−/−^ mice exposed to IFN‐γ loading (CD8a^−/−^‐rIFN‐γ mice) had markedly lowered blood perfusion and capillary density than CD8a^−/−^‐Saline mice (Figure 7A–D), which suggests that IFN‐γ lessened the angiogenesis in response to hypoxia. Moreover, as anticipated, IFN‐γ loading resulted in an increase in the levels of apoptotic cells and macrophages as well as the levels of MMP‐2 and MMP‐9 activities in the ischemic muscles of CD8a^−/−^ mice (Figure 7C,E,G,H), which indicates that CD8^+^ T‐cell activation negatively regulates the vascular regeneration associated with these cells' secretion of IFN‐γ in mice under our experimental hypoxic conditions.

### 3.6 IFN‐γ Depletion Facilitated Angiogenic Action in the Ex Vivo Experiment

An aortic ring culture assay has often been used to investigate ex vivo angiogenic responses [36]. As a next step to further examine our hypothesis, serum from CD8a^+/+^ mice that had undergone the ischemic surgery or sham operation was pre‐incubated on day 7 with saline (Sham‐7D‐Sal, Isch‐7D‐sal) or IFN‐γ neutralizing antibody (nIFN‐γ: Sham‐7D‐nIFN‐γ, Isch‐7D‐nIFN‐y, 400 ng/mL) for 30 min, respectively. The aortic rings of CD8a^+/+^ and IFN‐γ‐γ^+/+^ mice were cultured in the presence or absence of the four types of sera mentioned above, respectively, under normoxic and hypoxic conditions for 5 days. We observed that IFN‐γ depletion stimulated microtubule formation in both the wild‐type aortic rings from CD8a^+/+^ and IFN‐γ^+/+^mice (Figures S4C,D, S7B,C), which provided further evidence that CD8^+^ T cell‐derived IFN‐γ acts as a key mediator in angiogenic actions in vivo.

### 3.7 EGCG Promoted Ischemia‐Induced Angiogenesis in Mice

**FIGURE 8:**
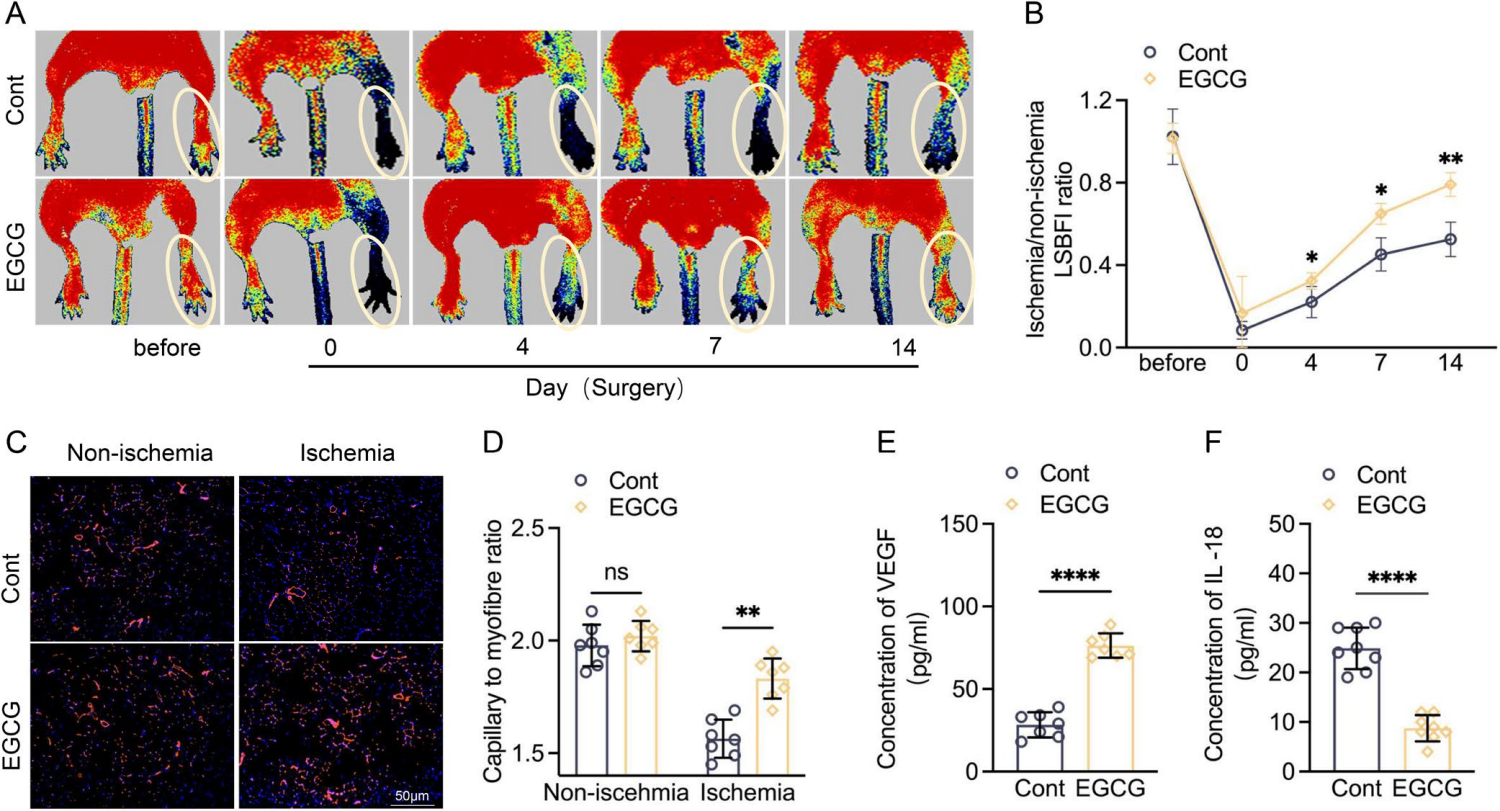
EGCG supplementation stimulated ischemic hindlimb blood flow recovery and capillary formation. (A, B) Representative LDBF images and quantitative data show the ischemia‐to‐nonischemia blood flow ratio in the ischemic hindlimbs of mice that received an intragastric administration of normal saline (Cont) and EGCG. (C, D) Immunofluorescence images and quantitative data showing the capillary density in ischemic and nonischemic thigh adductor muscles. (E, F) The ELISA results showing the serum levels of VEGF and IL‐18 in the two groups. Data are mean ± SEM (*n* = 7–8/group). Significance was assessed by a two‐way repeated measures ANOVA (B) or one‐way ANOVA (D) or unpaired Student's t test (E, F). *p < 0.05, **p < 0.01, ****p < 0.0001. Scale bar, 50 μm.

**FIGURE 9:**
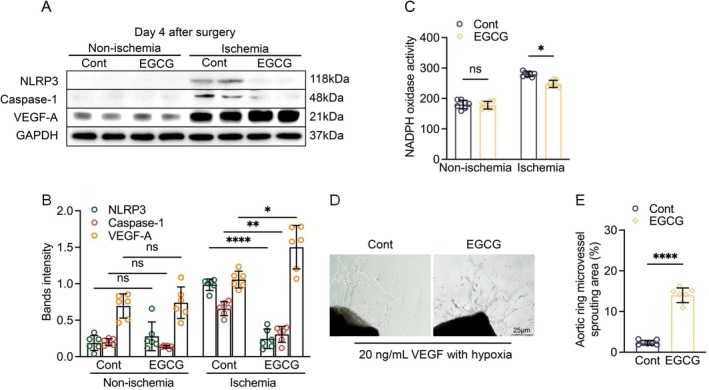
EGCG improved growth signal‐ and pyroptosis‐related protein changes. (A, B) Representative western blotting images and quantitative data for the levels of NLRP3, caspase‐1, and VEGF proteins in four groups (*n* = 6/group). (C) NADPH oxidase activity in the ischemic skeletal muscles of two groups (*n* = 7/group). (D, E) Aorta rings were cultured with 20 ng/mL of VEGF in the presence or absence of EGCG (20 μM) for 5 days. Representative images and quantitative data showing the aortic ring microvessel sprouting area (high magnification). The endothelial sprouting area is expressed as a percentage of pixels per image occupied by vessels in the quantitative area (*n* = 7/group). Data are mean ± SEM. Significance was assessed by one‐way ANOVA (B, C) or unpaired Student's t test (E). *p < 0.05, **p < 0.01, ****p < 0.0001. Scale bar, 25 μm.

**FIGURE 10:**
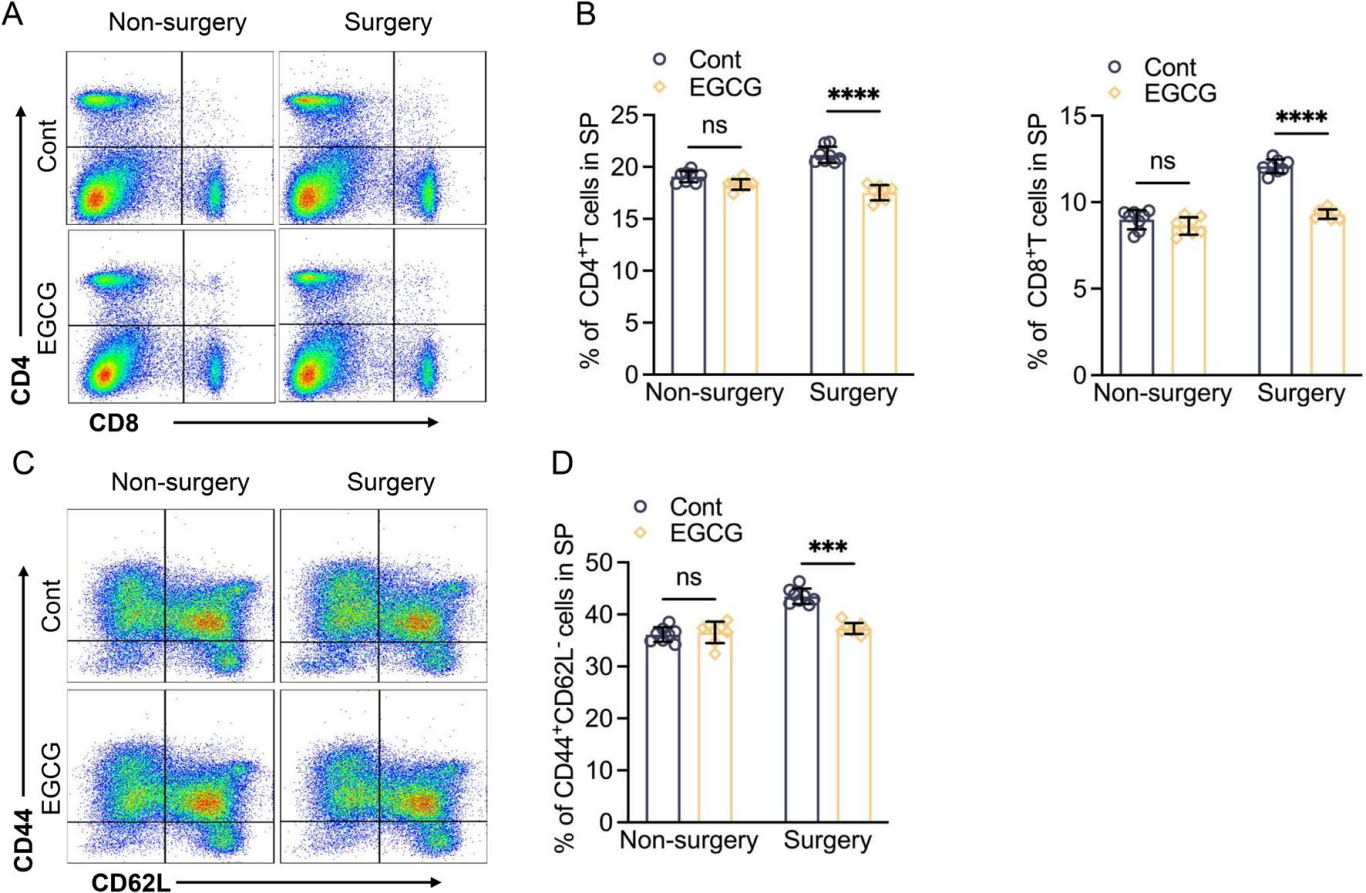
EGCG suppressed spleen CD4^+^ T cells, CD8a^+^ T cells, and CD44^high^CD64L^low^ effector memory CD8a^+^ T cells. (A, B) Representative FACS data showing the spleen CD4^+^ and CD8a^+^ T cells in the four groups. (C, D) Representative FACS data showing the CD44^high^CD64L^low^ effector memory CD8a^+^ T cell numbers in the four groups. Data are mean ± SEM (*n* = 8/group). Significance was assessed by one‐way ANOVA (C, D). ***p < 0.001, ****p < 0.0001.

EGCG treatment has been shown to produce a vascular benefit in humans and animals [37, 38]. Herein, CD8a^+/+^ mice were administered 200 μL of saline or EGCG solution (25 mg/kg/day) by gavage and then subjected to the related analyses. EGCG loading markedly increased the ischemic hindlimb blood flow and capillary density, accompanied by reductions in plasma IFN‐γ and IL‐18 levels and an elevation of VEGF (Figures 8 and S7D). Consistently, the EGCG treatment greatly ameliorated the harmful changes in the levels of NLRP‐3, caspase‐1, VEGF proteins, and NADPH oxidase activity of the CD8a^+/+^ mice compared to the non‐treated CD8a^+/+^ ischemic mice (Figure 9A–C). Moreover, EGCG loading lowered not only the levels of blood CD8a^+^ T cells and IFN‐γ, as well as the levels of spleen CD4^+^ T cells, CD8a^+^ T cells, and CD44^high^CD64L^low^ effector memory CD8a^+^ T cells (Figure 10) but also that of infiltrated CD8a^+^ T cells in ischemic muscle (Figures 10 and S7D–G). In the ex vivo CD8a^+/+^ aorta ring culture, EGCG stimulated VEGF‐induced microtubule formation under hypoxia (Figure 9D,E). These results, together with the qPCR data, demonstrated that EGCG treatment significantly suppressed the levels of MCP‐1, ICAM‐1, gp91^phox^, p22^phox^, MMP‐2/−9, and cathepsin S/K genes (Figure S8), suggesting that EGCG supplementation‐mediated vasculoprotection might be due to the reduction of CD8^+^ T cell‐derived IFN‐γ‐mediated inflammation, oxidative stress, apoptosis, and proteolysis.

### 3.8 EGCG Improved HUVEC Dysfunction in Response to 5% ATCM

We first tested the effects of the cultured medium from non‐activated and activated CD8^+^ T cells on HUVEC function. Compared to 5% NTCM, 5% ATCM impaired rhVEGF‐induced tubulogenesis, accompanied by elevations of NLRP‐3 and caspase‐1 proteins in HUVECs under hypoxia (Figure S9). Interestingly, EGCG improved 5% NTCM and hypoxia‐induced HUVEC migration, tubulogenesis, and apoptosis (Figure S10), indicating that EGCG can improve HUVEC dysfunction in response to 5% ATCM.

## 4 Discussion

Although immune cell‐mediated tissue repair has been an emerging paradigm in the field of regenerative medicine [39, 40], there is very limited evidence showing that adaptive immune cells such as cytotoxic lymphocytes contribute to tissue regeneration. In this study, we focused on the role(s) of the CD8^+^ T/IFN‐γ axis in ischemia‐induced angiogenesis in mice, and the most significant findings are that mice lacking CD8^+^ T cells were resistant to ischemic vascular injury and exhibited well‐developed capillary and blood flow recovery. At the cellular and molecular levels, CD8^+^ T deletion was shown to ameliorate harmful changes in the following ways: (1) decreasing the levels of plasma IFN‐γ, IL‐18, and NADPH oxidase activity and increasing plasma VEGF levels; (2) reducing the levels of oxidative stress (gp^91phox^ and p22^phox^)‐, inflammation (MCP‐1 and ICAM‐1), pyroptosis (NLRP‐3 and caspase‐1), and proteolysis (MMP‐2, MMP‐9, cathepsin K, and cathepsin S)‐related proteins and/or genes in the ischemic muscles; (3) elevating growth signal proteins (VEGF and p‐Erk1/2) of the ischemic muscles; and (4) reducing the numbers of infiltrated macrophages and CD8^+^ T cells in the ischemic muscles. The angiogenic actions were canceled by the administration of murine recombinant IFN‐γ both in vivo and ex vivo. All of these beneficial effects were reproduced by the IFN‐γ ablation. EGCG supplementation was also observed to exhibit a beneficial effect on vascular regeneration in CD8a^+/+^ mice under our experimental ischemic conditions. In vitro, EGCG improved 5% ATCM and hypoxia‐induced migration, tubulogenesis, and apoptosis accompanied by the reduction of NLRP‐3 and caspase‐1 levels in HUVECs.

The ability of ischemic stress to activate CD8^+^ T cells in blood and ischemic muscles is likely to contribute to the deterioration of neovascularization under our experimental conditions. Accumulating clinical and laboratory evidence suggests that the host immune system plays a pivotal role during tissue regeneration [39]. Recent investigations highlighted the roles of the adaptive immune cells as an essential player in the cardiac tissue healing process [41, 42]. T cells, particularly cytotoxic CD8^+^ T cells, were recently shown to modulate the repair and regeneration of various organ systems [42]. Our present findings demonstrated that CD8^+^ T‐cell deletion improved the recovery of ischemic hindlimb blood flow and capillary formation. The ischemic muscles of CD8a^−/−^ mice had increased levels of VEGF and Erk1/2 proteins. Because VEGF can promote angiogenesis in various pathobiologies, we propose that CD8^+^ T cells lessen the vascular regeneration in ischemic states through the negative modulation of VEGF‐Erk1/2 signaling in our experimental conditions.

It is well established that T cell‐derived IFN‐γ is harmful for various types of vascular and metabolic diseases by immune‐inflammation cross‐talk [9, 43]. In the present study, the ischemic stress markedly increased the levels of CD8^+^ T cells and macrophages as well as the levels of IFN‐γ, IL‐18, MCP‐1, ICAM‐1, NLRP3, and caspase‐1 in blood and/or ischemic muscles of CD8a^+/+^ mice; these changes were markedly reversed in CD8a^−/−^ mice. Comparable vasculoprotection and anti‐inflammation were reproduced in IFN‐γ^−/−^ mice, whereas these effects were almost completely diminished by the administration of IFN‐γ. CD80^+^ dendritic cell‐derived exosomes have been shown to suppress CD8^+^ T cells through a downregulation of NLRP3 expression in mice that underwent liver transplantation [44]. The upregulation of the IFN‐γ‐mediated immune‐inflammasome response could therefore represent a common mechanism in the deterioration of the vascular regeneration capacity in CD8a^+/+^ mice in response to ischemic stress. This concept was further supported by the ex vivo data of the aorta ring culture assay, i.e., 5% Isch‐7D‐nIFN stimulated microtubule formation in the aortic rings from CD8a^+/+^ and IFN‐γ^+/+^mice compared to the 5% Isch‐7D‐sal.

ROS can regulate angiogenesis, with NADPH oxidase being a major source of ROS in endothelial cells [45]. The results of the present experiments demonstrated that CD8a^−/−^ lowered NADPH oxidase activity and its membrane‐type component (*gp91* ^*phox*^ and *P22* ^*phox*^) gene expressions, which were associated with impaired endothelial angiogenic actions including proliferation, sprouting, migration, and tubulogenesis in vitro [46]. The apoptosis of various types of endothelial cells is sensitive to oxidative stress [19]. Our present observations revealed that CD8a^−/−^ significantly prevented apoptosis in the ischemic muscles. Similar to CD8a^−/−^, IFN‐γ^−/−^ yielded a vasculoprotective effect associated with the reduction of NADPH oxidase activity and apoptosis in vivo. IFN‐γ has been shown to induce endothelial apoptosis and physiological blood‐vessel regression in response to tumor ischemia [47]. Collectively, these data provide evidence that the deterioration of vascular regeneration in CD8a^+/+^ mice is mediated, at least in part, by the reduction of oxidative stress‐mediated apoptosis which was mediated by the CD8^+^/IFN‐γ axis in mice under our experimental conditions.

The ability of proteases (i.e., MMPs and cathepsins) to cleave extracellular matrix proteins and growth factors is known to contribute both negatively and positively to vascular regeneration [48]. One of our research group's earlier investigations demonstrated that MMP‐2 deficiency impaired ischemia‐induced neovascularization via the modulation of mature endothelial functions and endothelial progenitor cells' mobilization in young and aged mice [33]. Conversely, recombinant MMP‐2 has been shown to induce membrane‐bound CD100 shedding from CD8^+^ T cells and soluble CD100 generation, resulting in an enhancement of CD8^+^ T‐cell cytotoxicity toward endothelial cells in patients with acute myocardial infarction [49].

Among the members of the cathepsin family, cathepsin S inhibited angiogenesis via the release of a potent anti‐angiogenic protein, i.e., endostatin from the C terminus of collagen XVIII [50]. We observed herein that not only CD8a^−/−^ but also IFN‐γ^−/−^ can produce a vascular benefit accompanied by reductions of infiltrated macrophages and MMP‐2, MMP‐9, cathepsin S, and cathepsin K activities and/or gene levels in mouse ischemic muscles. Accumulating evidence indicates that inflammatory cytokines and oxidative stress stimulate these protease expressions in macrophages [48]. Thus, the upregulation of proteolytic activity in macrophages by inflammatory cytokines and oxidative stress might also contribute to the deterioration of vascular regeneration in CD8a^+/+^ mice.

We next evaluated the efficacy of EGCG in ischemic hindlimb mice. In part because of its cost‐effective and efficacious properties, green EGCG has been widely studied. As the most abundant catechin in green tea, EGCG is regarded as a potent antioxidant and plays positive roles in various aspects of human physiology such as anti‐inflammation, anti‐apoptosis, and autophagy promotion [51]. EGCG significantly attenuated histopathologic changes (vitiligo) in the skin by reducing excessive inflammatory responses, especially the infiltration of CD8^+^ T cells, and by markedly inhibiting inflammatory mediators such as tumor necrosis factor‐alpha (TNF‐α) and IL‐6 levels [52]. After the administration of EGCG to the hindlimb ischemic CD8a^+/+^ mice in this study, we observed improved vascularization, increased plasma VEGF levels, reduced oxidative stress production, and a reduced inflammatory response including the levels of circulating CD8a^+^ T‐cell, IFN‐γ, and IL‐18 levels and spleen CD8a^+^ T‐cell and CD44^high^CD64L^low+^ effector memory CD8a^+^ T cell; the levels of NLRP3, caspase‐1, MCP‐1, and ICMA‐1 proteins and/or genes; and the numbers of infiltrated CD8a^+^ T cells in the ischemic muscles. The in vitro data indicated that EGCG improved the 5% ATCM‐induced HUVEC migration, tubulogenesis, and apoptosis associated with the reductions of NLRP3 and caspase‐1. Taken together, these findings suggest that EGCG supplementation facilitates vascular regeneration in ischemic states through a positive modulation of VEGF signaling, inflammation, oxidative stress, apoptosis, and proteolysis in mice under our experimental conditions.

Several limitations of this work should be considered. First, we could not use specific CD8^+^ T‐cell IFN‐γ deficiency (IFN‐γ^f/f^; Cd8a‐Cre^+^) mice to obtain direct evidence regarding whether CD8^+^ T cell‐derived IFN‐γ works as a key negative modulator in ischemia‐induced angiogenesis in mice. Second, we could not fully identify the cell source of IFN‐γ during the ischemic angiogenesis process in the CD8a^+/+^ and IFN‐γ^+/+^ mice. Third, we did not perform adoptive CD4^+^ and CD8^+^ T‐cell therapies to explore both types of T‐cell function in angiogenesis in the CD8a^−/−^ and IFN‐γ^−/−^ mice. Finally, it is necessary to explore how the CD8^+^ T‐cell/IFN‐γ axis regulates VEGF‐Erk1/2 signaling in vivo and in vitro.

Taken together, our findings describe an unappreciated role of CD8^+^ T cells in the pathogenesis of PAD. We obtained evidence of CD8^+^ T‐cell negative regulation of vascular regeneration by IFN‐γ secretion in our experimental conditions. The EGCG‐mediated vasculoprotective effect might be associated with the modulation of the CD8^+^ T‐cell/IFN‐γ‐immune‐inflammation‐oxidative stress‐apoptosis axis overaction. Our findings offer new avenues for developing novel therapeutics targeting CD8^+^ T cells in ischemic peripheral artery diseases.

## Author Contributions

X. Piao conceived the project, designed and performed experiments, implemented and analyzed the data, interpreted data, prepared figures, and drafted and wrote the article. J. Jin, L. Zhao, Y. Lei, Y. Li, M. Narisawa, S. Shu, X. Yue, and J. Piao participated in the experiments and performed the blinded data analysis of immunostaining. L. Dai performed Network Pharmacology Analysis. C. Zhu, L. Hu, and Q. Cui discussed the results and revised and critically reviewed the article. X.W. Cheng conceived the project, designed and performed experiments, interpreted data, and revised and critically reviewed the article. All authors read and approved the final version of the article.

## Ethics Statement

All animal experimental procedures were performed according to the Guide for the Care and Use of Laboratory Animals published by the US National Institutes of Health and approved by the Institutional Animal Care and Use Committee of Yanbian University (protocol no: YD202309110014).

## Consent

The authors have nothing to report.

## Conflicts of Interest

The authors declare no conflicts of interest.

## Supporting information


**Data S1:** Supporting Information.

## Data Availability

The data that support the findings of the animal study are available from the corresponding author upon reasonable request. Source data are available online in the public database as described in the Network Pharmacology Analysis.
