# Supplementary material for: CD8 + T Cells Negatively Modulate Ischemia‐Induced Angiogenesis in Mice
Source: FASEB J. 2025 Nov 17;39(22):e71165. doi: 10.1096/fj.202503027R (PMC12621164; doi:10.1096/fj.202503027R)

CD8$^+$ T-Cell Negative Modulates Ischemia-Induced Angiogenesis in Mice

***Corresponding author:**

Prof. Xian Wu Cheng, Department of Cardiology and Hypertension, Yanbian University Hospital, 1327 Juzijie, Yanji, Jilin PR. 133000, China. Electronic address: chengxw0908@163.com

Supplemental Materials

Tables S1

Figures S1-S10

Suppl Table S1. Primer sequences used for the quantitative real-time PCR in the mice

Genes	Forward Primers	Reverse Primers
gp91^{phox}	ACTTTCCATAAGATGGTAGCTTGG	GCATTCACACACCACTCAACG
p22^{phox}	AACTACCTGGAGCCAGTTGAG	AATTAGGAGGTGGTGGAATATCGG
Cathepsin S	GTGGCCACTAAAGGGCCTG	ACCGCTTTTGTAGAAGAAGAAGGAG
Cathepsin K	AGCAGGCTGGAGGACTAAGGT	TTTGTGCATCTCAGTGGAAGACT
ICAM-1	CCCCGCAGGTCCAATTC	CCAGAGCGGCAGAGCAA
MCP-1	GCCCCACTCACCTGCTGCTACT	CCTGCTGCTGGTGATCCTCTTGT
MMP-9	CCAGACGCTCTTCGAGAACC	GTTATAGAAGTGGCGGTTGT
MMP-2	CCCCATGAAGCCTTGTTTACC	TTGTAGGAGGTGCCCTGGAA
GAPDH	ATGTGTCCGTCGTGGATCTGA	ATGCCTGCTTCACCACCTTCT

Suppl Table S1. Primer sequences used for the quantitative real-time PCR in the mice. GAPDH: glyceraldehyde 3-phosphate dehydrogenase, ICAM-1: intercellular adhesion molecule-1, MCP-1: monocyte chemoattractant protein-1, MMP: matrix metalloproteinase, PCR: polymerase chain reaction.

Supplementary Figure legends

Suppl. Fig. S1. The in vivo experimental protocols. **Exp1:** The protocol used for the $CD8^{-/-}$. **Exp2:** The protocol used for the IFN-$\gamma^{-/-}$ mice. **Exp3:** The protocol used for the $CD8^{-/-}$ mice loaded with murine recombinant IFN-γ. **Exp4:** The protocol used for the $CD8^{+/+}$ mice supplemented with EGCG (25 mg/kg/day).

Suppl. Fig. S2. CD8 deficiency reduced the infiltration of macrophages and the MMP-2 and MMP-9 activities in the ischemic muscles. **A,B:** Representative immunofluorescence images and quantitative data showing the numbers of infiltrated macrophages in ischemic and nonischemic muscles of two groups (n=7/group). **C,D:** Representative gelatin zymography images and quantitative data for the MMP-2 and MMP-9 gelatinolytic activities in ischemic and nonischemic muscles of the two groups (n=4/group). Data are mean ± SEM. Significance was assessed by one-way ANOVA (B,D). *p<0.05, **p<0.01, ***p<0.001. Scale bar, 50 μm.

Suppl. Fig. S3. $CD8^{+}$ deletion lowered the targeted molecule gene expressions in the ischemic muscle. **A**–**D:** qPCR data showing the levels of MCP-1, ICAM-1, gp91phox, p22phox, MMP-2, MMP-9, cathepsin S, and cathepsin K. Data are mean ± SEM (n=7/group). Significance was assessed by one-way ANOVA (A–D). **p<0.01, ***p<0.001, ****p<0.0001.

Suppl. Fig. S4. CD8 deficiency reduced apoptosis in the ischemic muscles. **A,B:** Representative immunostaining images and quantitative data showing the $ssDNA^{+}$ numbers in the ischemic and nonischemic muscles of two groups. **C,D:** Aorta ring culture results assay showing the sprouting microtube of $CD8^{+/+}$ aortas in the presence of four types of sera (5% Sham-7D-Sal, 5% Sham-7D-nIFN, 5% Isch-7D-sal, and 5% Isch-7D-nIFN) for 5 days. Data are mean ± SEM (n=7/group). Significance was assessed by one-way ANOVA (B,D). *p<0.05, **p<0.01. Scale bars, 50 μm (A) or 25 μm (C).

Suppl. Fig. S5. IFN-γ deficiency reduced apoptosis and MMPs' activities in the ischemic muscles. **A,B:** Representative gelatin zymography images and quantitative data for the MMP-2 and MMP-9 gelatinolytic activities in ischemic and nonischemic muscles of two groups (n=4/group). **C,D:** Representative immunostaining images and

quantitative data showing the $ssDNA^+$ numbers in the ischemic and nonischemic muscles of two groups (n=7/group). **E:** Quantitative data show the numbers of the infiltrated macrophages in in the ischemic and nonischemic muscles of two groups (n=7/group). Data are mean ± SEM. Significance was assessed by one-way ANOVA (B,D). *p<0.05, ****p<0.0001. Scale bar, 50 μm.

Suppl. Fig. S6. IFN-$\gamma^{-/-}$ lowered the targeted molecule gene expressions in the ischemic muscle. **A–D:** qPCR data showing the levels of MCP-1, ICAM-1, gp91phox, p22phox, MMP-2, MMP-9, cathepsin S, and cathepsin K. Data are mean ± SEM (n=7/group). Significance was assessed by one-way ANOVA (A–D). **p<0.01, ***p<0.001, ****p<0.0001.

Suppl. Fig. S7. EGCG suppressed circulating $CD8^+$ T cells and IFN-γ levels in $CD8^{+/+}$ mice. **A:** ELISA data showing the levels of serum IFN-γ in four groups. **B,C:** Representative microscopies and quantitative data of aorta ring assay showing the sprouting microtubes of IFN-$\gamma^{+/+}$ aortas in the presence of two types of sera (5% Isch-7D-sal and 5% Isch-7D-nIFN) for 5 days. **D:** ELISA data showing the serum IFN-γ levels in the two groups. **E,F:** Representative and quantitative FACS data showing the circulating $CD8^+$ T cells in the four groups. **G,H:** Representative immunofluorescence images and quantitative data showing the $CD8^+$ T-cell numbers in the ischemic muscles of the two groups. Data are mean ± SEM (n=7–8/group). Significance was assessed by one-way ANOVA (A,E) or unpaired Student's t-test (C,D,G). ****p<0.0001. Scale bars, 25 μm (B), 50 μm (F).

Suppl. Fig. S8. EGCG administration lowered the targeted molecule gene expressions in the ischemic muscle. **A–D:** qPCR data expressing the levels of MCP-1, ICAM-1, gp91phox, p22phox, MMP-2, MMP-9, cathepsin S, and cathepsin K. Data are mean ± SEM (n=7/group). Significance was assessed by one-way ANOVA (A–D). ***p<0.001, ****p<0.0001.

Suppl. Fig. S9. Activated $CD8^+$ T-cell condition medium (ATCM) impaired the rhVEGF-induced tubulogenesis in HUVECs under hypoxic stress. **A,B:** 5% ATCM suppressed microtubule formation in HUVECs under hypoxic conditions (n=10/group). **C,D:** Representative western blotting images and quantitative data for the levels of

NLRP3 and caspase-1 proteins in two experimental groups (n=6/group). Significance was assessed by unpaired Student's t-test (B,D). ***p<0.001, ****p<0.0001. Scale bar, 50 μm.

Suppl. Fig. S10. EGCG improved the 5% ATCM- and hypoxia-induced HUVEC migration, tubulogenesis, and apoptosis. **A,B:** Representative microscopy results and quantitative data showing the numbers of migrated HUVECs in three groups (5% NTCM, 5% ATCM, and 5% ATCM+EGCG20μM). **C,D:** Representative microscopy results and quantitative data showing the microtubule length in the three groups as above. **E,F:** Representative and quantitative FACS data showing the numbers of apoptotic cells in the three groups. Data are mean ± SEM (n=8-10/group). Significance was assessed by one-way ANOVA (B,D,E). ***p<0.001, ****p<0.0001. Scale bars, 20 μm (A), 50 μm (C).

Supplement Figure S1

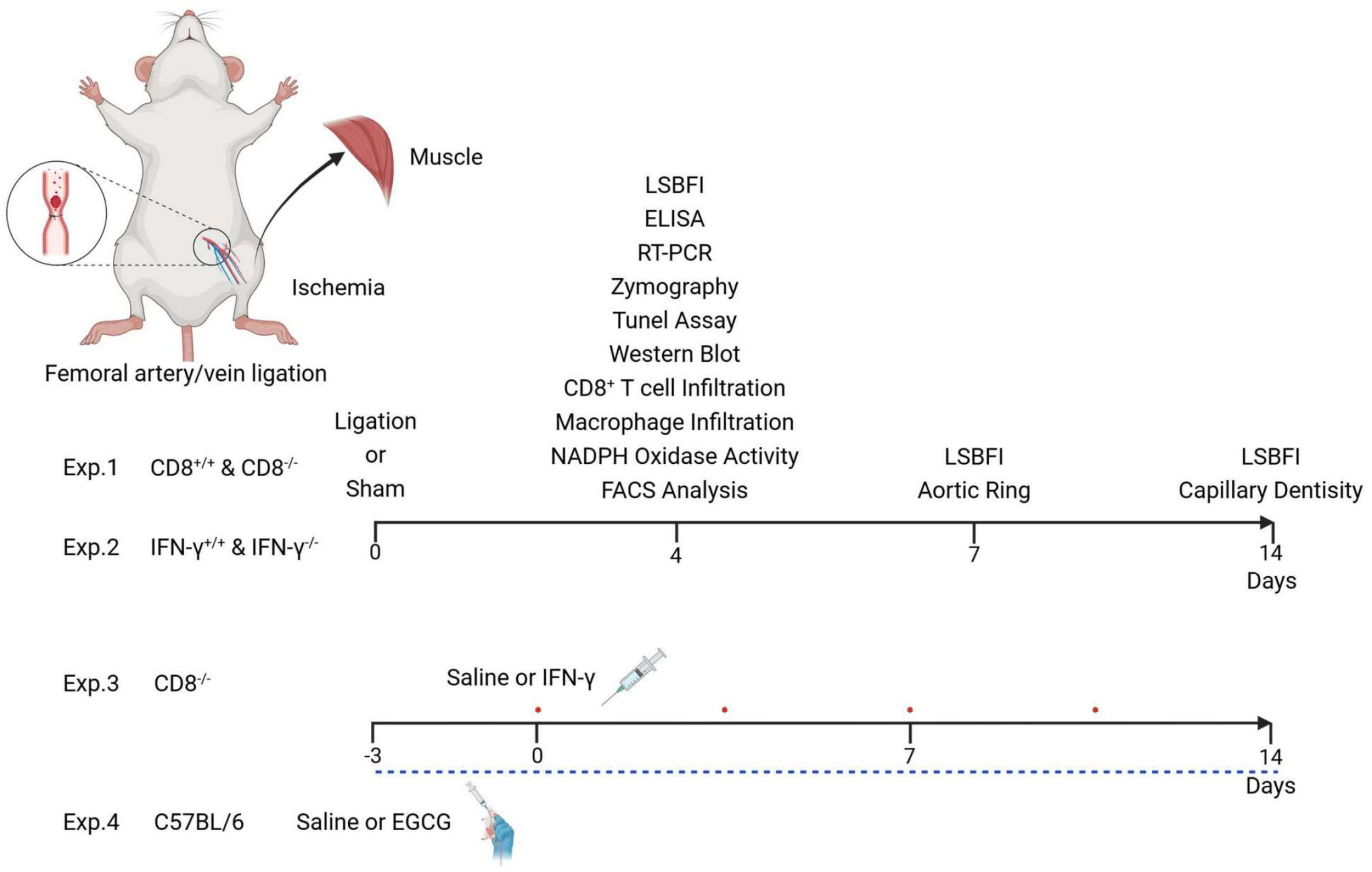

Supplement Figure S2

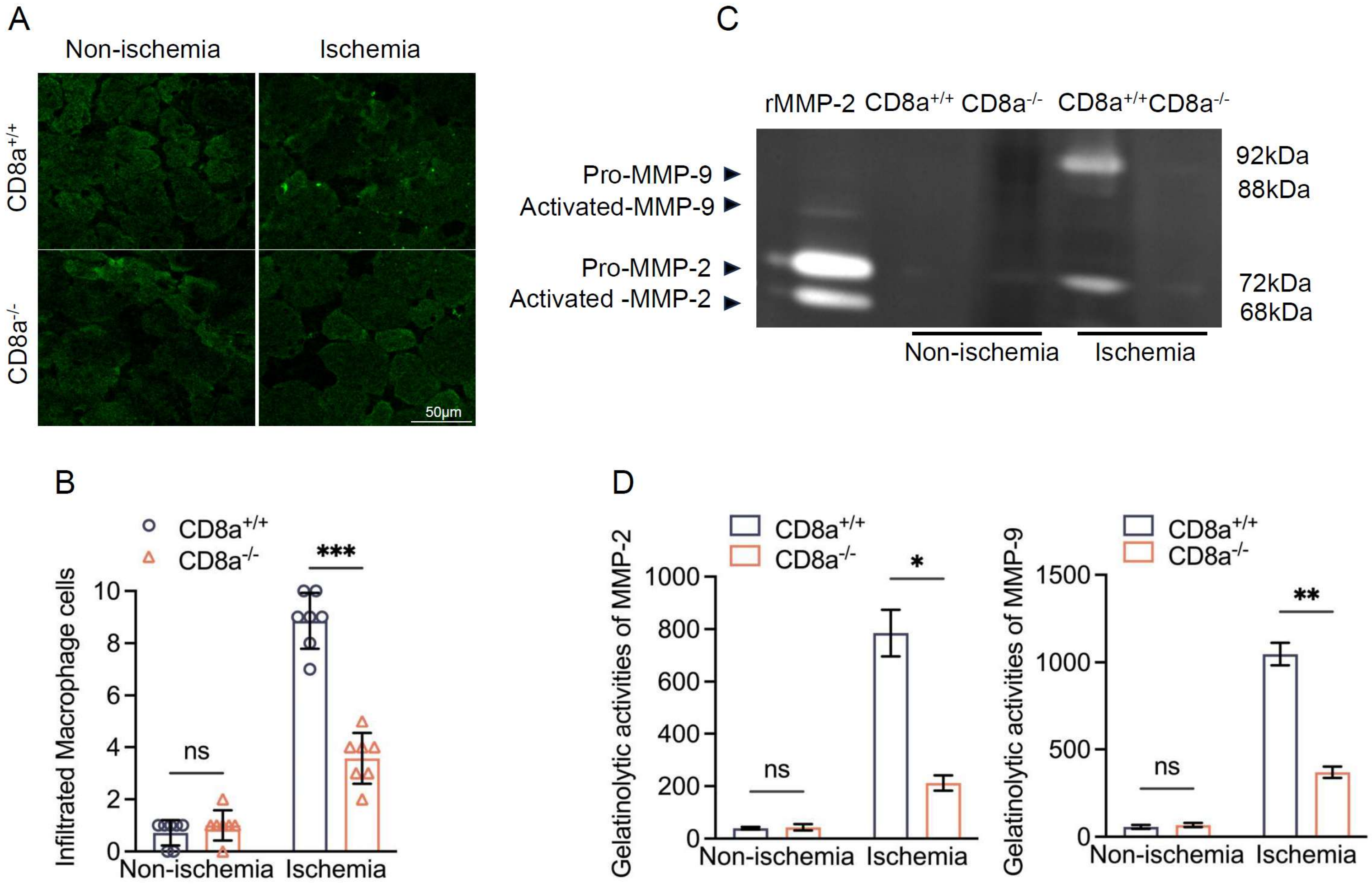

Supplement Figure S3

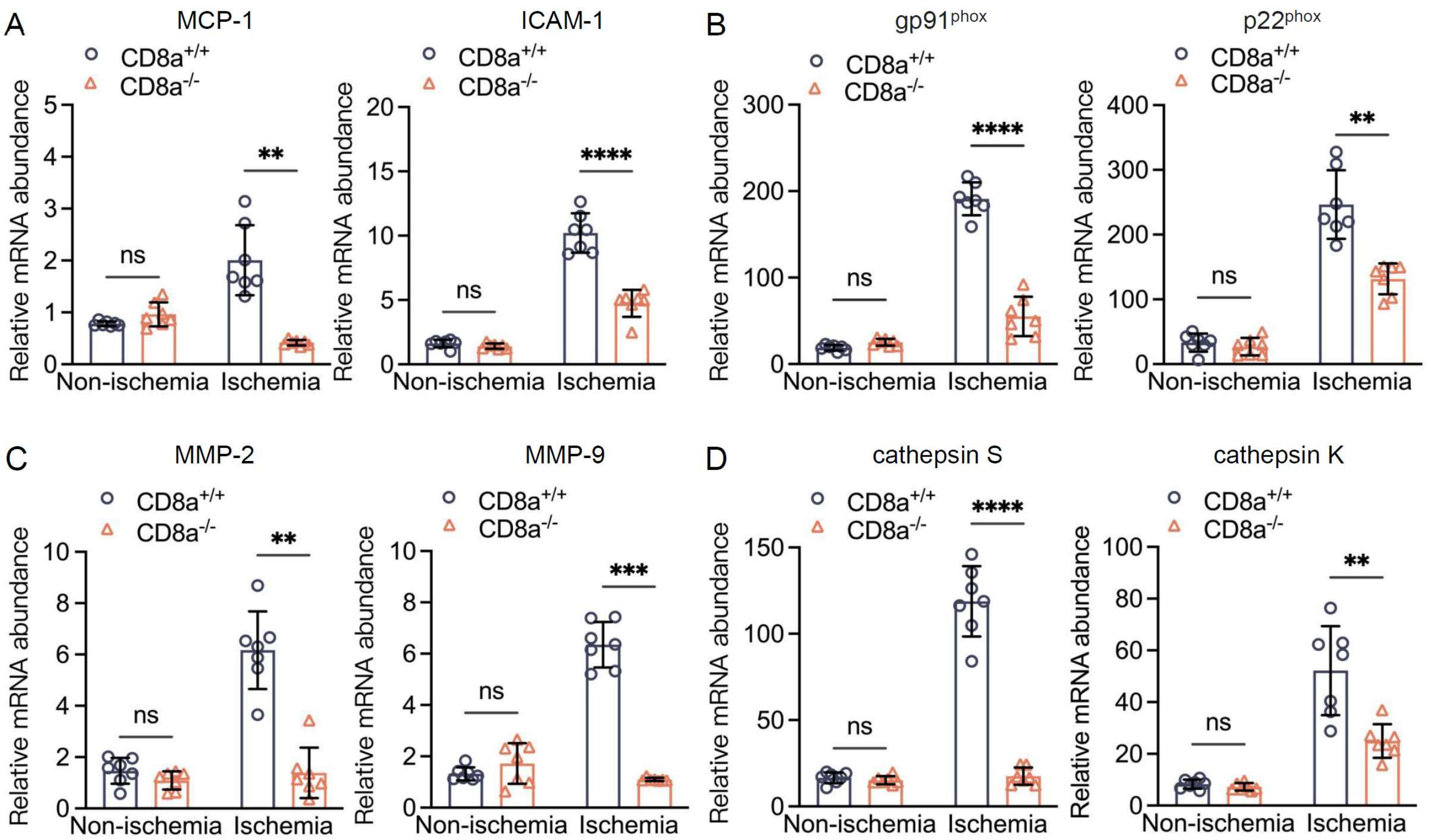

Supplement Figure S4

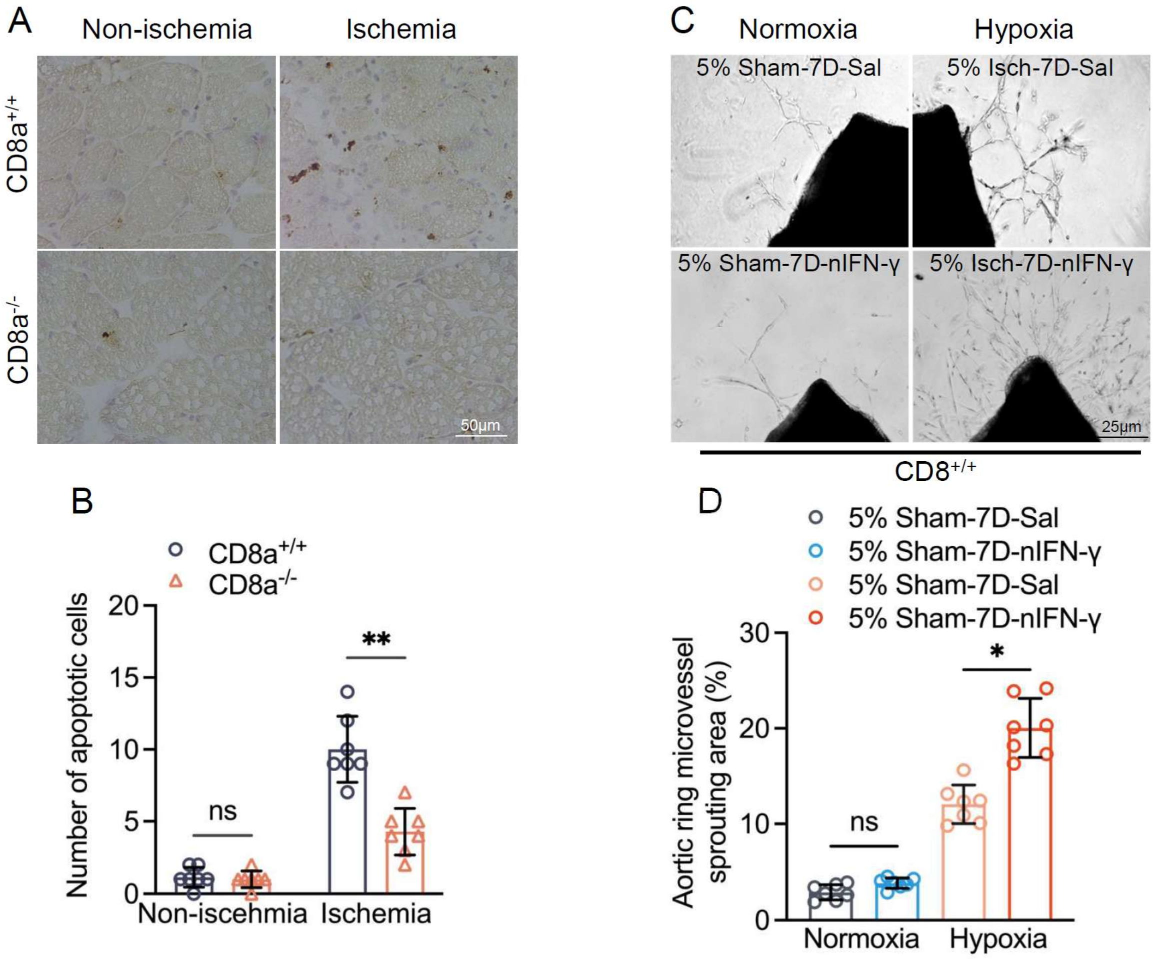

Supplement Figure S5

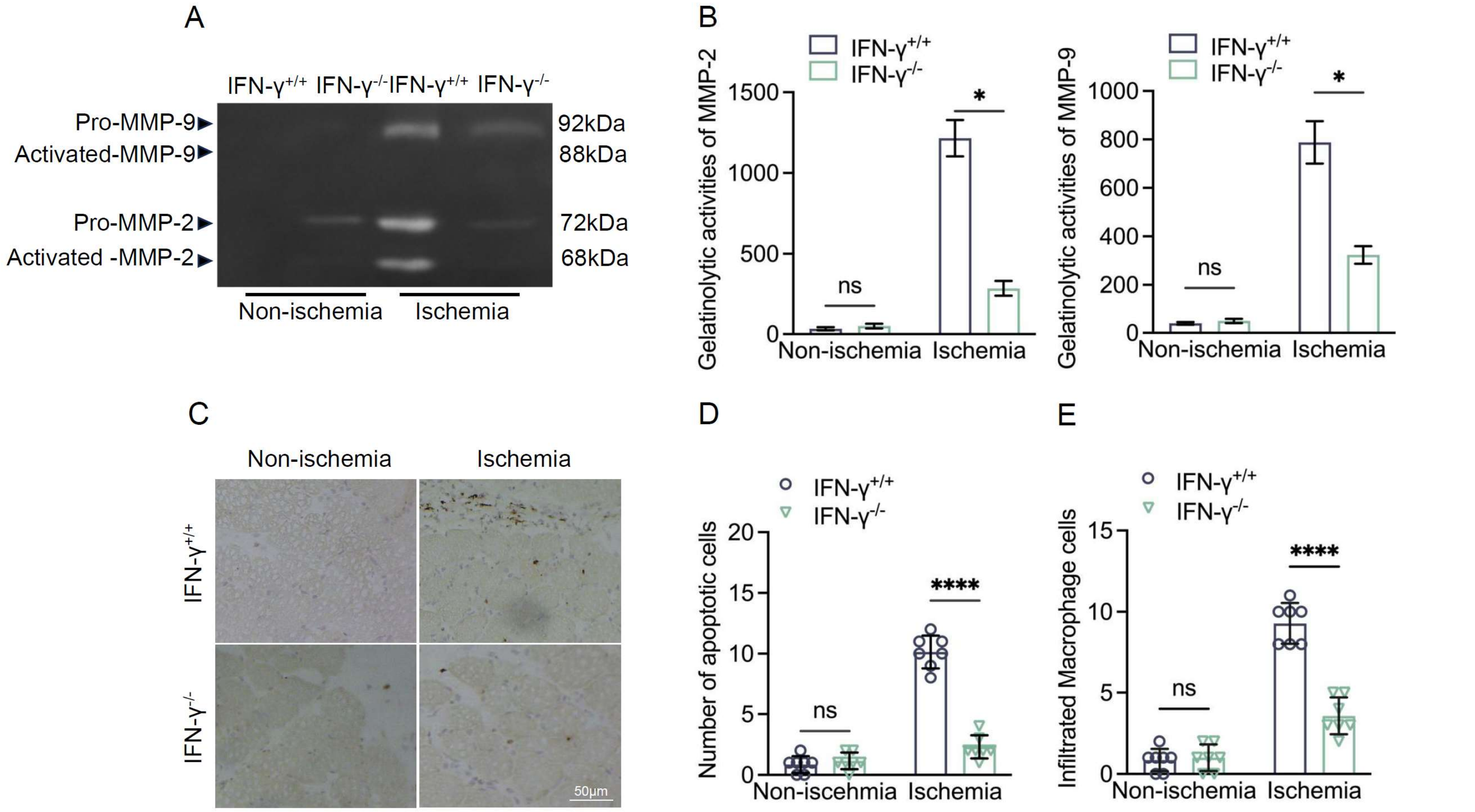

Supplement Figure S6

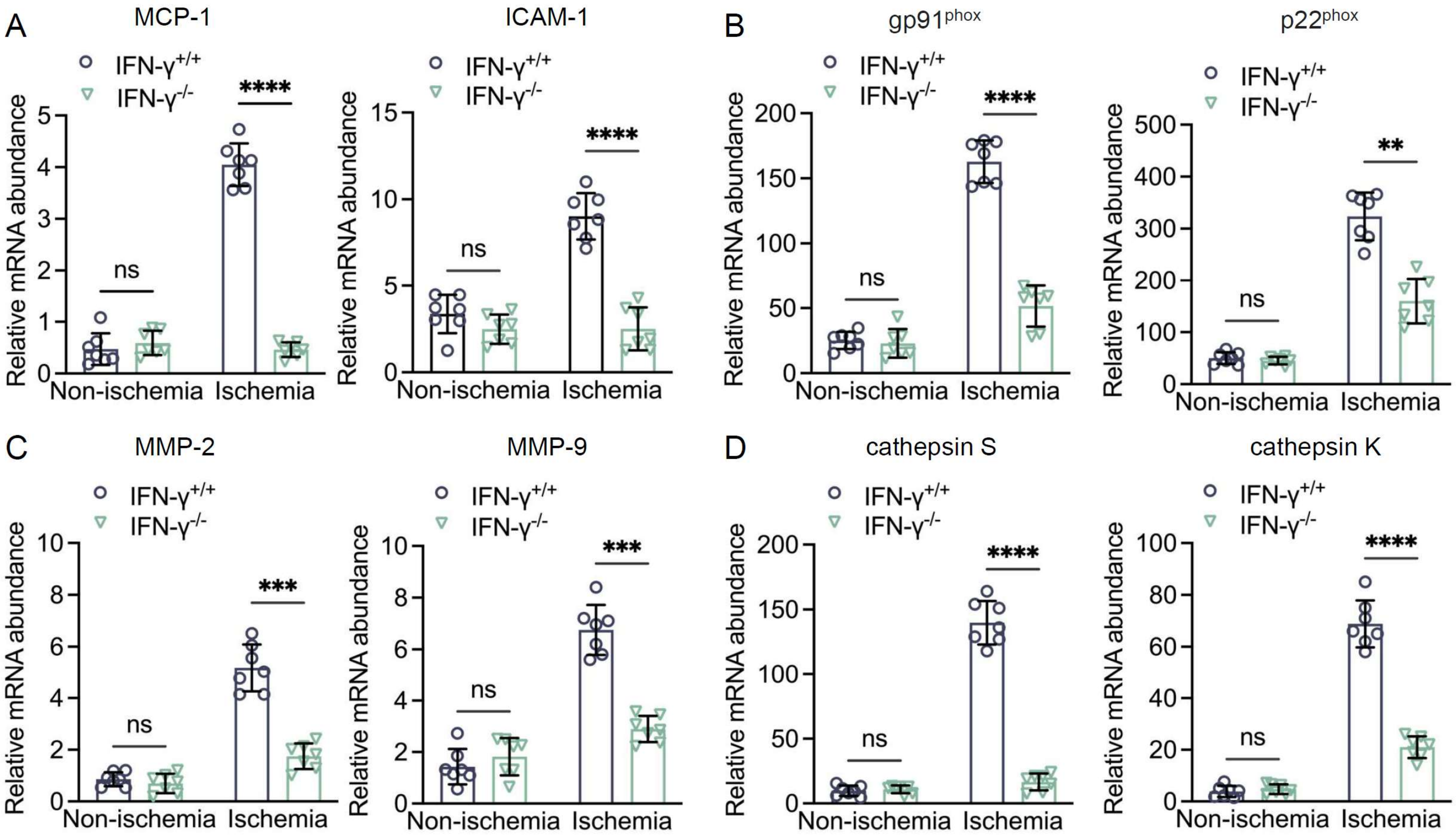

Supplement Figure S7

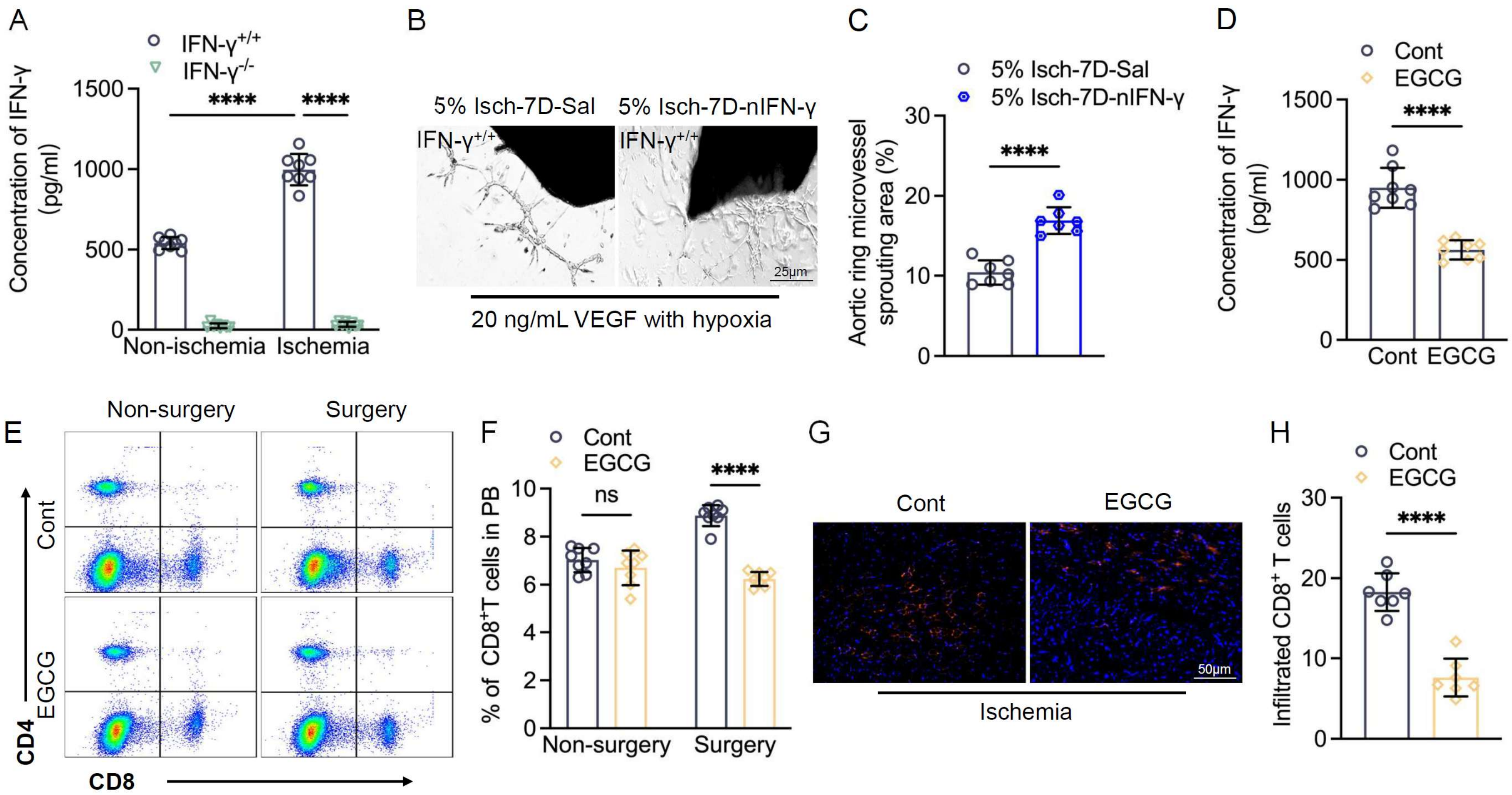

Supplement Figure S8

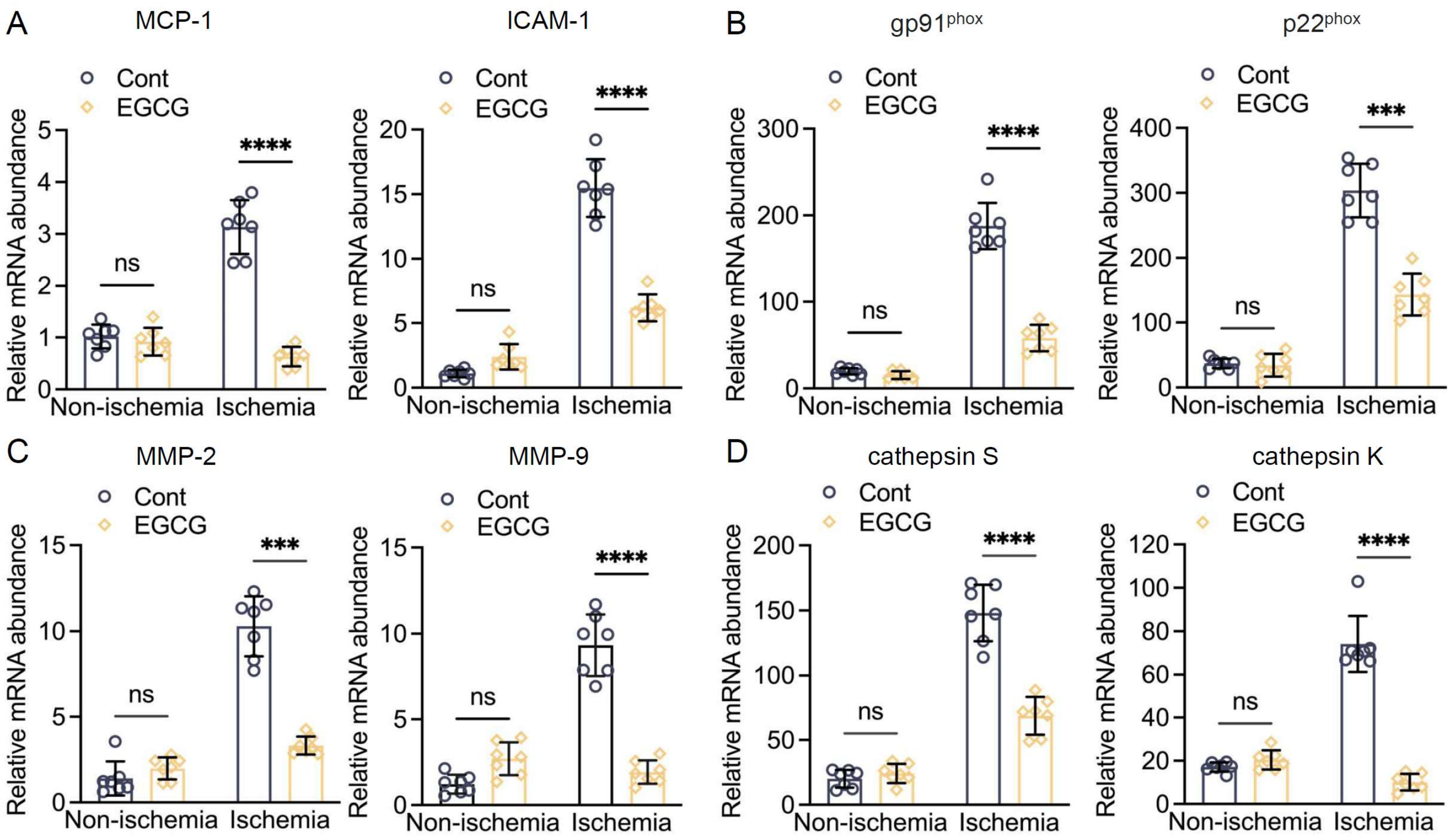

Supplement Figure S9

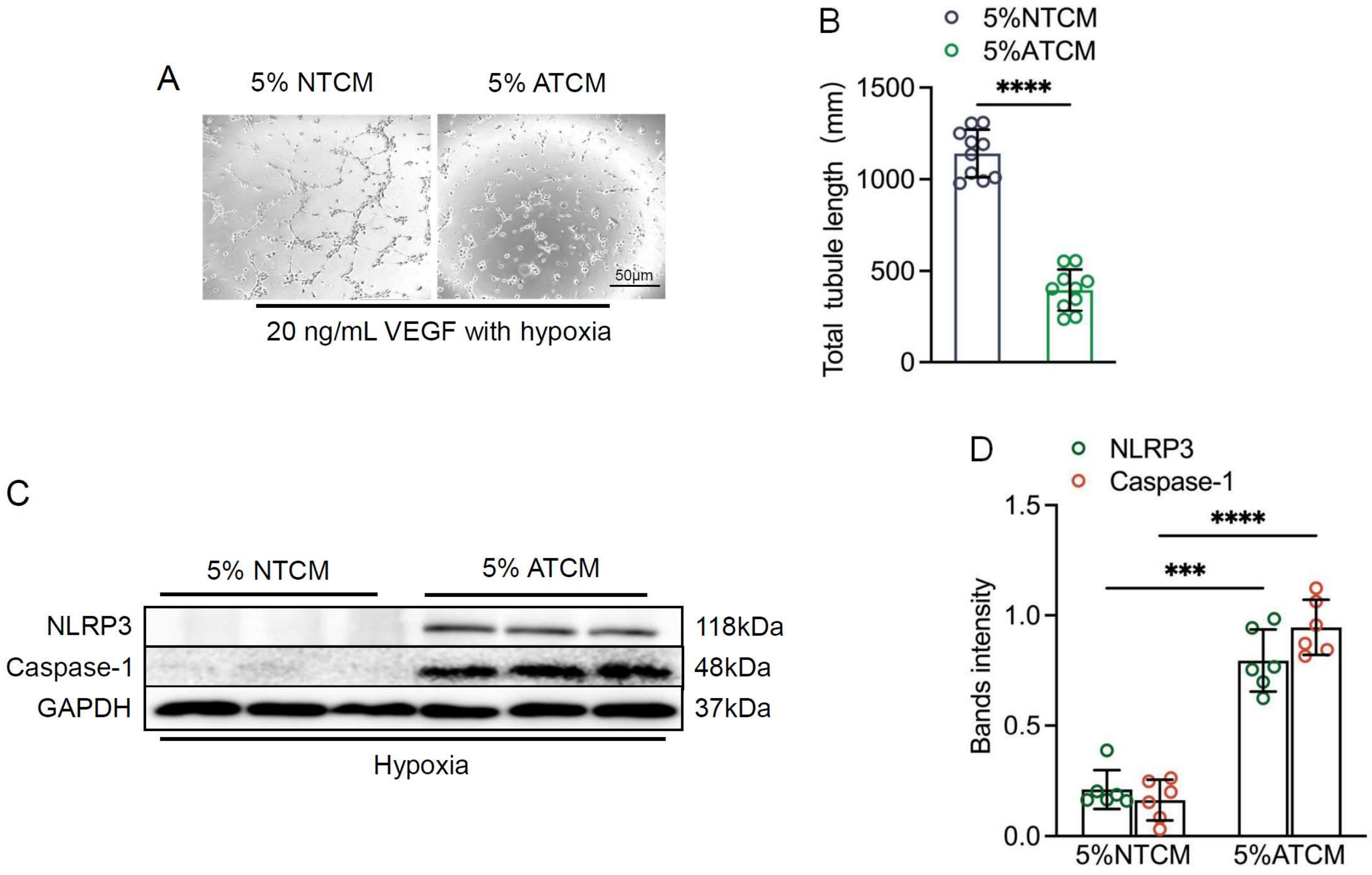

Supplement Figure S10

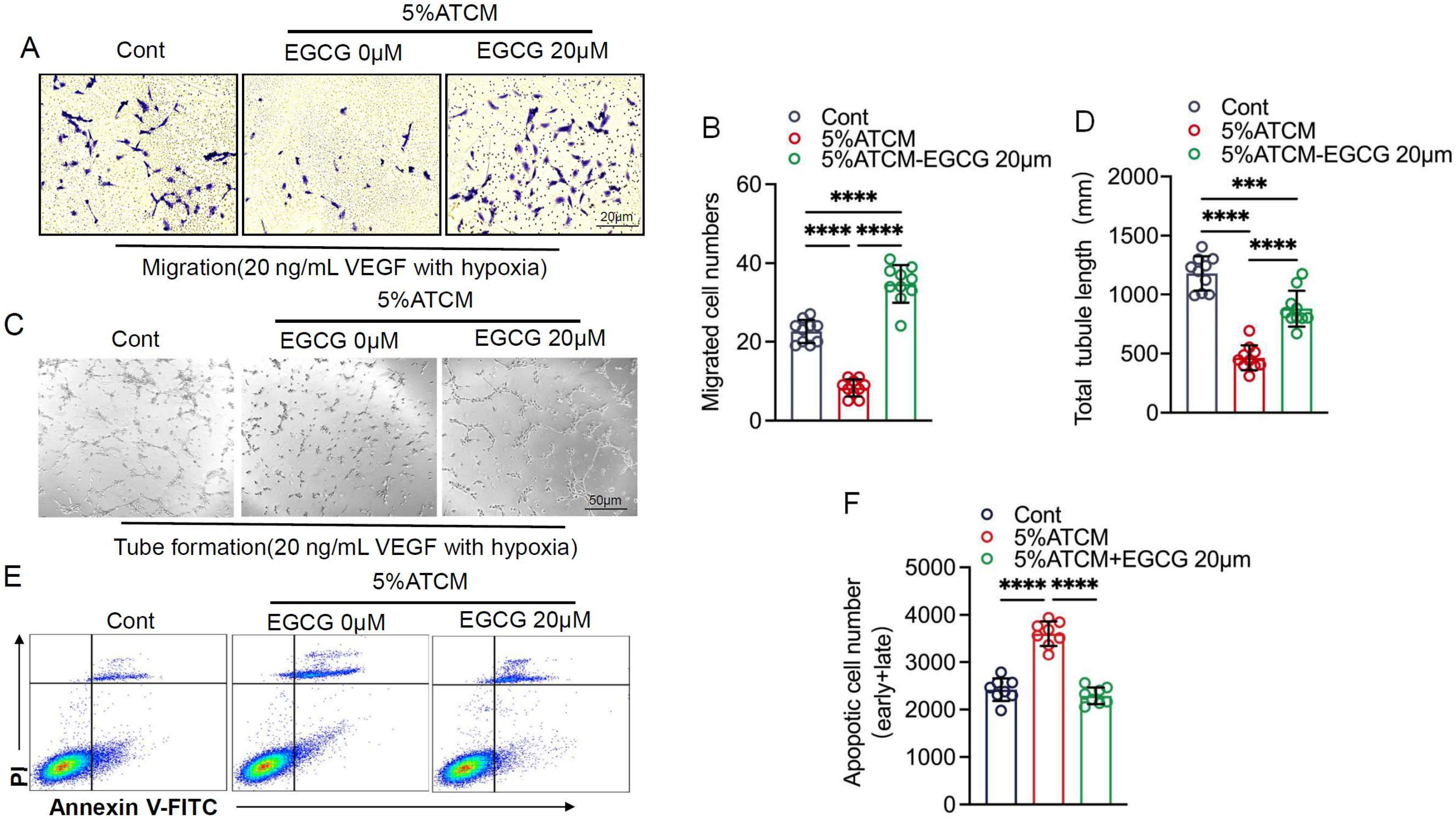

Supplement: Supplementary file 1 — Data S1: Supporting Information. [file FSB2-39-e71165-s001.pdf]
